# Detecting comorbidity patterns in rare disease patients with machine learning

**DOI:** 10.3389/fepid.2026.1765678

**Published:** 2026-05-04

**Authors:** Benjamin Mark Connor, Claire Hill, Lu Bai, Amy Jayne McKnight, Anna Jurek-Loughrey

**Affiliations:** 1 School of Electronics, Electrical Engineering and Computer Science, Queen’s University Belfast, Belfast, United Kingdom; 2 School of Medicine, Dentistry and Biomedical Sciences, Centre for Public Health, Queen’s University Belfast, Belfast, United Kingdom

**Keywords:** comorbidity, machine learning, multimorbidity, pattern detection, rare disease

## Abstract

**Introduction:**

Whilst individually rare, affecting only a small percentage of the population, rare diseases as a whole impact around 6% of the global population (with this number likely an underestimate). Rare diseases are often complex, with specific challenges in diagnosis, management, and treatment due to limited knowledge and research. Rare disease patients have been shown to have more comorbidities compared to those without a rare disease diagnosis. Studying comorbidities in patients with rare diseases is particularly important as these patients may exhibit unique patterns of multiple diseases which are not well understood. Understanding these comorbidity patterns can lead to insights into the etiology and progression of rare diseases, potentially identifying new therapeutic targets and improving clinical management strategies. Additionally, studying comorbidities can help in predicting complications, improving the quality of life of patients, and offering a more comprehensive approach to health care for those affected by rare diseases.

**Methods:**

A machine learning based method known as hierarchical clustering was applied to diagnosis data from the UK Biobank to study comorbidity patterns in patients with rare diseases. The results were then compared with patterns detected for the general population.

**Results:**

Twelve clusters were identified for the rare disease group, and 14 for the no rare disease group.

**Discussion:**

Unique comorbidity patterns were observed for individuals with and without a rare disease diagnosis, highlighting potential priorities for intervention to improve both disease management and patient care.

## 1 Introduction

In the European Union, a rare disease (RD) is defined as any disease that affects no more than 1 person in 2000. However, RDs are only rare when considered in isolation; with over 6,000 RDs recognised by Orphanet [1], RDs collectively have an estimated global prevalence of 3.5%–5.9%, equating to around 263–446 million people affected at any one time [2]. The burden of RDs is pronounced further as most RDs are genetic and exclusively exhibit paediatric onset, meaning patients suffer for a prolonged period and receive treatment for a vast portion of their lives. Despite being an important area of clinical focus, the low incidence of RDs individually means that many healthcare practitioners may never see or diagnose a given RD in their lifetime; however, they are likely to encounter patients with a RD diagnosis of some kind. It is imperative that we seek to broaden our understanding of these conditions in a general sense, with the aim of guiding more informed healthcare decisions for patients diagnosed with a RD. A popular method used to further the understanding of diseases is the study of disease comorbidity, defined as the co-occurrence of two or more health conditions in an individual [3]. Research on the comorbidities of RDs in a general sense is, however, extremely limited, and hence will be the focus of this study.

Comorbidity significantly affects patient well-being: patients experiencing numerous diseases concurrently are more likely to report a poor self-rated health status, especially when combined with a chronic condition [4]. Experiencing comorbid conditions complicates the treatment plan for concerned individuals and is a driving factor in the shift of modern healthcare practice to a precision medicine strategy [5] (as opposed to the traditional disease-centered approach). Individuals with a rare disease have been shown to have a significantly higher total number of chronic diseases compared with patients without a rare disease [6].

Recent analysis of comorbidity data falls into two main categories: predictive analysis and pattern analysis. Predictive analysis has been used extensively to predict contraction of a new comorbid condition given an individual with an existing chronic disease[7–12], and to predict mortality given a patient with a specific combination of comorbid conditions [9, 13]. The primary goals of pattern analysis conducted on comorbidity data are identification of phenotypes, and the identification of clinically relevant subgroups of patients based on their comorbidity patterns. Unsupervised Machine Learning techniques can to organise information so that heterogeneous data can be classified into relatively homogeneous groups. These groups can then be analysed, and novel conclusions drawn to further understanding of the dataset. Hierarchical clustering [14] has been commonly used to identify patient subgroups in different applications. Its key advantage is that patients with similarities can be grouped together, without the need for assignment of the desired number of clusters prior to analysis—the number of clusters is determined by the results of the analysis.

Often analyses are carried out on patient populations diagnosed with an “index” disease—a disease whose diagnosis is chosen as a prerequisite for consideration in an analysis. Most studies focus on a chronic illness, as these are long-term ailments that require continual treatment, and may interact unexpectedly with subsequently diagnosed comorbid conditions. Cluster analysis techniques have been shown to be effective in identifying clinically relevant groups of patients with an index disease according to their comorbidities [15–22]. Analyses are either carried out on a combination of clinical features and comorbidity data, or on comorbidity data exclusively.

Cluster analysis has also been shown to be effective at identifying clinically relevant comorbidity subgroups in much broader cohorts of patients. These studies face different challenges to that of the index disease studies as they generally cover much larger datasets with many more features, introducing issues with computational complexity, the “curse of dimensionality” (distance between data points becoming meaningless), and noise [23]. Several studies analyse patients in primary care and have demonstrated that chronic diseases appear in clusters, even among patients who don’t share an index disease [24–26]. Commonly, comorbidity data is reduced to a binary representation (1 for present, 0 for not present), and a form of analysis chosen. Hierarchical cluster analysis [25], multiple correspondence analysis [27], and exploratory factor analysis (EFA) [28] have all been supported as effective when applied to binary representations of comorbidity data. Studies of primary care patients carried out at a smaller, local scale further promote hierarchical clustering as being effective in combination with binary diagnosis data [29–31]. Combination methods of deep-learning based latent space learning and traditional clustering methods have also been shown to be effective for identifying comorbidity patterns [32, 33]. **Motivation:** Pattern analysis has been widely used to identify disease co-occurrence structures both in cohorts defined by an index condition and in the general population. Such analyses have informed healthcare planning, risk stratification, and clinical guideline development by revealing previously unrecognized comorbidity structures. Understanding how diseases cluster is therefore essential not only for optimizing service provision, but also for improving insight into shared pathophysiological mechanisms and patient trajectories. However, despite the growing body of research on comorbidity, RDs remain largely underrepresented in comorbidity pattern analyses. Most existing studies focus on common chronic conditions (e.g., diabetes or cardiovascular disease), where large patient numbers facilitate pattern discovery. While a small number of studies have explored comorbidities within individual RDs, systematic analyses examining comorbidity patterns across rare diseases as a group are lacking. Moreover, comparative analyses evaluating whether comorbidity structures in RD populations differ from those observed in the general population have not, to our knowledge, been reported. This represents an important gap. Patients with RDs often experience delayed diagnosis and fragmented care pathways. Identifying characteristic comorbidity patterns in this population may therefore have direct implications for earlier detection of complications, improved care coordination, and hypothesis generation regarding shared biological pathways. The present study addresses this unmet need by systematically analysing comorbidity patterns across RDs and directly comparing them with patterns observed in the general population, thereby providing novel insight into the comorbidity landscape of RD patients. **Contribution:** Using data from the UK Biobank, this study has identified shared patterns of comorbidity between rare and more common diagnoses, such as specific patient sub-groups experiencing gastrointestinal diseases and joint disorders, female reproductive and birth conditions, and benign neoplasms. Unique patterns of comorbidity in both RD and non-RD were also observed. For the RD group, these include patient subgroups experiencing urinary and male genital disorders, malignant neoplasia, and self-harm, poisoning or mental disorders. This work highlights potential focus points for future research to understand the RD burden, provide targeted interventions and improve patient quality of life.

## 2 Materials and methods

### 2.1 Data

The data used for this research was taken from the UK Biobank, a large-scale biomedical database and research resource containing genetic, lifestyle and health information from half a million UK participants aged 40–69 (at recruitment), recruited between 2006 and 2010 [34]. Rare disease has been previously mapped in the UK Biobank [35]. This is the first study to map comorbidity patterns and compare between RD and NRD. A repeat assessment of participants was made between 2012 and 2013. The data contains a multitude of features for each patient record, both categorical and continuous, and across multiple points in time. Disease diagnosis information is available for each patient until the end of 2021.

Disease diagnosis fields were extracted for analysis (Biobank fields 41270); these take the form of a list of ICD-10 codes attributed to each patient record. Data field 41,270 relates to distinct diagnosis codes present for a participant which have been recorded across all hospital inpatient records in either the primary or secondary position. This data is available for 448,651 UK Biobank participants [as shown in UK Biobank Showcase [36]]. Individuals with no ICD-10 diagnosis code (*n* = 62,385) were excluded from the present study as as no diagnosis codes were available for clustering in downstream pipelines. Following this, records were split into two categories: those with at least one RD diagnosis (*n* = 211,866), and those with no rare disease (NRD) diagnosis (*n* = 228,108). This was achieved through extraction of rare disease ICD-10 codes obtained from the Orphanet knowledge base [37] (June 2022 version). Each record’s list of ICD-10 codes was checked against the list of rare ICD-10 codes from Orphanet. Those with at least one RD ICD-10 code were put into the RD category, and the remainder placed into the NRD category. As there are more than 14,000 unique ICD-10 codes, each code was truncated to its 3-character parent and categorised according to UK Biobank Level 1 categorisation in order to reduce the number of features to a manageable level. Each category was converted to a column header and binary-encoded according to disease absence (0) or presence (1) for each patient record, resulting in 263 features, each corresponding to a disease category. e.g., “A00-A09 Intestinal infectious diseases.”

For each cohort, disease categories that were prevalent in less than 1% of records for that dataset were excluded. The aim was to avoid skewing clustering results by way of creating many outliers. The most common diagnosis category (Z80-Z99: “Persons with potential health hazards related to family and personal history and certain conditions influencing health status”) was present in more than 50% of the both datasets and so was disregarded to avoid skewing results, as well as having questionable clinical relevancy. A total of 114 features were removed from the RD category, and 168 features from the NRD category. The final dataset format is shown in Table 1. A full list of the disease categories for each cohort is available as Supplementary Table S1.

**Table 1:** Summary of the dataset used in the comorbidity study. “No. of Features” corresponds to the number of binary-encoded disease categories used for analysis after removal of low-prevalence categories.

Dataset	No. of records	No. of features
Rare disease diagnosis (RD)	211,866	149
No rare disease diagnosis (NRD)	228,108	95

### 2.2 Methods

An overview diagram of the analysis pipeline used in this comorbidity study is available as Supplementary Figure S1. The pipeline takes as an input two sub-datasets, representing records of patients with and without RD respectively. It then runs in three steps, outlined by coloured boxes in Supplementary Figure S1, followed by result analysis and validation. All analyses were carried out using Python version 3.10.5, numpy version 1.26.4, pandas version 1.16.0, scipy version 1.12.0, scikit-learn version 1.4.0, umap-learn version 0.5.5, matplotlib version 3.8.2 and seaborn version 0.13.2. A Github repository containing all code used for the analysis is available at: https://github.com/B-M-Connor/Detecting-Comorbidity-Patterns-in-Rare-Disease-Patients-with-Machine-Learning

1. **Dimensionality reduction:** High-dimensional data spaces often suffer from the “curse of dimensionality” [23], where the distance between data points becomes less meaningful as the number of dimensions increases. This makes it challenging to identify relevant subgroups of data points (i.e., records). In this study, each possible disease category is converted to a binary feature with value of 1 indicating disease presence for that record, and a value of 0 indicating disease absence for that record. After pre-processing, there are 149 features and 211,866 records in the RD sub-dataset, and 95 features and 228,108 records in the NRD sub-dataset. The data space is therefore very large. In such spaces, points tend to become equidistant from one another, diminishing the effectiveness of distance-based clustering algorithms [38]. In order to address this challenge, we performed dimensionality reduction for the RD and NRD records. Uniform Manifold Approximation and Projection (UMAP) [39] is a novel dimensionality reduction technique that has evidence of being effective in the context of identifying patterns in patient comorbidity with a similar number of binary-encoded features [17]. UMAP was presented [39] as an alternative to the popular t-SNE method [40], offering increased speed and better preservation of the global structure of the dataset [39], meaning it is well suited to large datasets such as the UK Biobank data. UMAP was applied to the two datasets, reducing them to a varying specified number of resultant dimensions 5, 10, 20, 30, 40, 50, 60 (NRD only), 75 (RD only). Each of these results converts the subject dataset from its original number of features to a UMAP embedding; this is a lower-dimension numeric representation of the data with the same number of rows, and the specified number of resultant dimensions (features). As there were 95 features in the NRD group prior to dimensionality reduction, a smaller maximum number of 60 dimensions instead of the 75 used for the RD group. 60 is close to the original number of dimensions, and would not have provided effective reduction. The UMAP algorithm has a “minimum distance” parameter, which determines how close together (or similar) unique records can be in the reduced space. As the aim was density, which will create cleaner separations between clusters, the minimum distance was set to 0. This means very similar records may be reduced by UMAP to being exactly the same, which helps when the data is being clustered. To maintain the global structure of the large dataset, a nearest-neighbours parameter value of 200 was chosen. Smaller values constrain UMAP’s learning to a more localised region, which in a large dataset such as the UK Biobank would likely lead to loss of the data’s underlying structure. A value larger than this begins to increase the computation time and complexity, again exacerbated by the large nature of the UK Biobank dataset. Values in the range 3 to 250 were tested and evaluated through manual inspection of resultant visualisations. The default Euclidean distance metric was maintained, in line with current literature. This results in 7 unique UMAP embeddings for the RD group, and 7 unique UMAP embeddings for the NRD group, each with varying number of dimensions.
2. **Agglomerative hierarchical cluster analysis:** Agglomerative hierarchical cluster analysis [41] is a machine learning based bottom-up clustering technique that initially places each data point in its own cluster, and iteratively merges similar clusters according to a linkage metric. This process repeats until one final cluster containing all of the data points is attained. The algorithm can be “cut off” at any point in the process according to a pre-ordained number of clusters chosen by the user. Pairwise distance matrices were calculated using the Euclidean distance metric for each of the previous UMAP embeddings of varying dimensions, for each of the two datasets. Cluster analyses with numbers of resultant clusters in the range 5–15 were carried out on the RD distance matrices, and resultant clusters in the range 10–15 for the NRD distance matrices. The range 5-15 was chosen as the number of clusters that is manageable in a clinical context for the RD group. The range 10–15 was chosen for the NRD group to keep results in a comparable range. The “average” cluster linkage metric was chosen after inspection of current literature in a similar context [17]. Under this approach, the distance between two clusters is calculated as the arithmetic mean of all pairwise distances between observations in one cluster and those in the other cluster. The cluster analysis was carried out with varying number of resultant clusters on each of the 7 UMAP embeddings generated for both the RD and NRD subgroups. This produced a total of 77 clustering results for the RD group, and 42 clustering results for the NRD group.
3. **Model selection:** The average silhouette coefficient (SC) [42] was calculated for each of the obtained clustering results to identify the optimal combination of UMAP dimensions and number of resultant clusters for each of the two datasets. Silhouette coefficient provides a score between −1 and 1 for each sample in the cluster; −1 indicates that a sample has been placed in the wrong cluster, 0 indicates clusters are overlapping, and 1 indicates the clusters are perfectly defined and distinctive. The model setup (i.e., UMAP dimension and cluster number) with the highest average silhouette score across clusters for each dataset was chosen for further analysis. The formula used to calculate SC and further details on its interpretation are provided in the Supplementary Material.
4. **Result analysis and validation:** It is important to note that the analyses in this study are based on unsupervised machine learning, specifically clustering. Since there are no predefined labels or outcomes, the cluster quality and robustness were evaluated using internal metrics, primarily the average Silhouette score, which measures how well each sample fits within its assigned cluster relative to other clusters. This approach allows us to assess the consistency and separability of the identified comorbidity patterns without relying on labelled data. The implemented analysis pipeline provided two sets of clusters as an output (i.e., record groupings), one for the RD records and one for NRD records. The output groupings were analysed to obtain insights as actionable as possible. The prevalence of each disease category within each cluster was calculated and compared against its prevalence in the full dataset. The disease categories with the greatest positive difference in prevalence (i.e, more prevalent in the cluster than in the wider dataset) were reported to give an initial understanding of the cluster’s key characteristics. Due to the limited body of research specifically addressing comorbidity patterns in rare disease populations, direct comparison of our findings with existing studies was not feasible. While comorbidity has been extensively investigated in common chronic conditions and general population cohorts, analogous analyses focused on rare diseases remain scarce. For validation of the clustering based approach, the most prevalent disease categories within each cluster extracted from the RD group were chosen for analysis using Relative Risk (RR) and Phi Coefficient (PHI), two traditional methods for analysing comorbidity across a dataset. For each pair of diseases, we constructed a 2 × 2 contingency table from the patient-level binary comorbidity matrix. Relative Risk (RR) was used to quantify the directional excess probability of one disease given the presence of another. 95% confidence intervals were calculated for RR using the log-Wald normal approximation method, as advised by the UK Department for Health and Social Care [43]. If 1 is not within the confidence interval, the RR score can be said to be statistically significant. Phi coefficient (PHI) was computed as a symmetric measure of association strength between binary diagnoses. RR values > 1 imply increased risk in that direction, and positive Phi coefficient values imply association between two diagnoses (similarly to correlation). Together, these metrics were used to validate the comorbidity patterns discovered in our study. The formulas used to calculate RR and PHI values and details on their interpretation are provided in the Methodology section of the Supplementary Material.

## 3 Results

### 3.1 Data demographics

For privacy reasons, all minimum and maximum age values provided are the mean of the top and bottom five values in each case. There is a minimum age across the entire dataset of 38, and maximum age of 72. The mean age across the entire dataset is 57. The age distribution skews toward the older end of the range; the 60–65 range contains the highest number of records, followed by the 65–70 range, and then the 55–60 range. There are very few records in both the 35–40 and 70–75 age ranges. The dataset has a female majority (54.8% female); there are 241,224 females and 198,750 males. The mean BMI across the entire dataset is 27.56, and ethnicity is distributed as follows: 94.18% White, 1.94% Asian/Asian British, 1.58% Black/Black British, 0.58% Mixed, 1.71% other.

**Figure 1:**
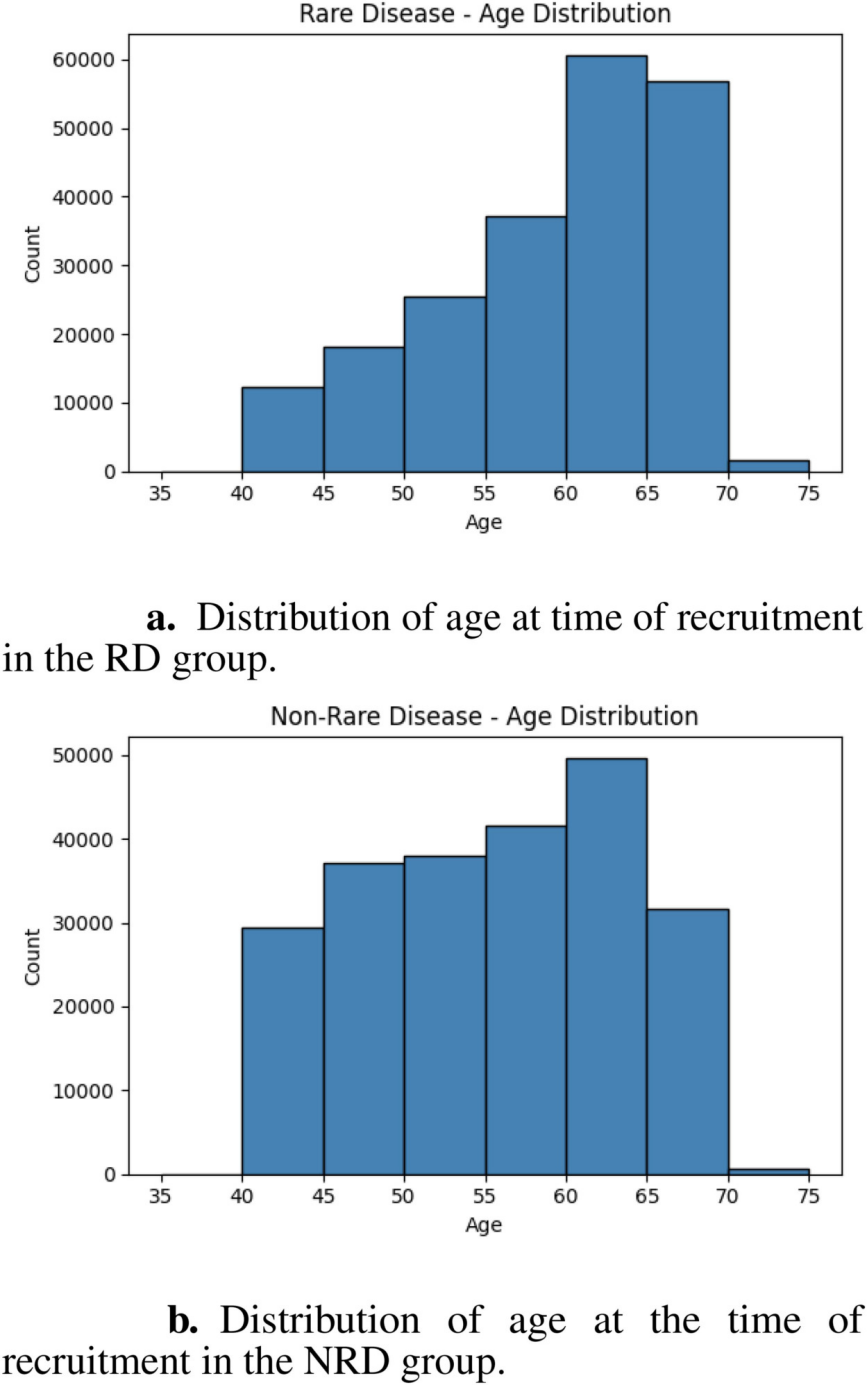
Age distributions for the RD and NRD groups. **(a)** distribution of age at time of recruitment in the RD group. **(b)** distribution of age at the time of recruitment in the NRD group.

The age distribution for the RD group is shown in Figure 1a. Within the RD sub-dataset, the minimum age is 39 and maximum age is 72, with a mean of 59. The RD sub-dataset is 49% male and 51% female. The age distribution for the NRD group is shown in Figure 1b. Within the NRD sub-dataset, the minimum age is 39 and maximum age is 71, with a mean of 55.

The NRD sub-dataset is 41% male and 59% female. Table 2 contains a summary of demographic information between the two groups. Table 3 contains summary diagnostic information between the two groups. Tables with summary diagnostic information broken down by age, sex and ethnicity are available as Supplementary Tables S2–S4.

**Table 2:** Summary demographics of the RD and NRD groups.

Group	Rare disease (RD)	No rare disease (NRD)
Mean age	58.94	55.11
% Female	50.73	58.63
% Male	49.27	41.37
Mean BMI	28.10	27.06
% White	94.22	94.15
% Mixed	0.50	0.65
% Asian/Asian British	2.11	1.79
% Black/Black British	1.51	1.65
% “Other”	1.71	1.76

**Table 3:** Summary diagnostic information for the RD and NRD groups.

Group	Rare disease (RD)	No rare disease (NRD)
Median Charlson Comorbidity Index	3	1
Median no. of diagnoses	17	5
Top 10 Diagnoses (no. of times diagnosed)	I10: Essential (Primary) hypertension (104,725) Z86.4: Personal history of psychoactive substance abuse (54,508) K44.9: Diaphragmatic hernia without obstruction or gangrene (38,059) K57.3: Diverticular disease of large intestine without perforation or abscess (35,773) Z92.2: Personal history of long-term (current) use of other medicaments (35,548) Z86.7: Personal history of diseases of the circulatory system (32,411) E11.9: Non-insulin-dependent diabetes mellitus without complications (30,736) K21.9: Gastro-oesophageal reflux disease without oesophagitis (29,466) J45.9: Asthma, unspecified (28,988) I25.1: Atherosclerotic heart disease (28,842)	I10: Essential (Primary) hypertension (46,286) K57.3: Diverticular disease of large intestine without perforation or abscess (20,895) Z86.4: Personal history of psychoactive substance abuse (18,525) J45.9: Asthma, unspecified (17,554) K44.9: Diaphragmatic hernia without obstruction or gangrene (14,720) H26.9: Cataract, unspecified (12,975) M17.9: Gonarthrosis [arthrosis of knee], unspecified (12,567) Z88.0: Personal history of allergy to penicillin (12,107) K21.9: Gastro-oesophageal reflux disease without oesophagitis (11,162) R07.4: Chest pain, unspecified (11,157)
Top 5 Rare Diagnoses (no. of times diagnosed)	C44.3 & C44.4: Pilomatrix carcinoma (16,140) C50.9: Hereditary breast and/or ovarian cancer syndrome (7,360) D70: Congenital neutropenia (7,085) C34.9: Pleuropulmonary blastoma (3,617) C67.9: Non-papillary transitional cell carcinoma of the bladder (3,090)	N/A

### 3.2 Model selection

Details on the selection process for the optimal clustering model are available as Supplementary Material. Heatmap plots of the silhouette scores attained for varying number of clusters and UMAP dimensions are available as Supplementary Figure S2.

### 3.3 Model analysis

#### 3.3.1 Rare disease model

**Figure 2:**
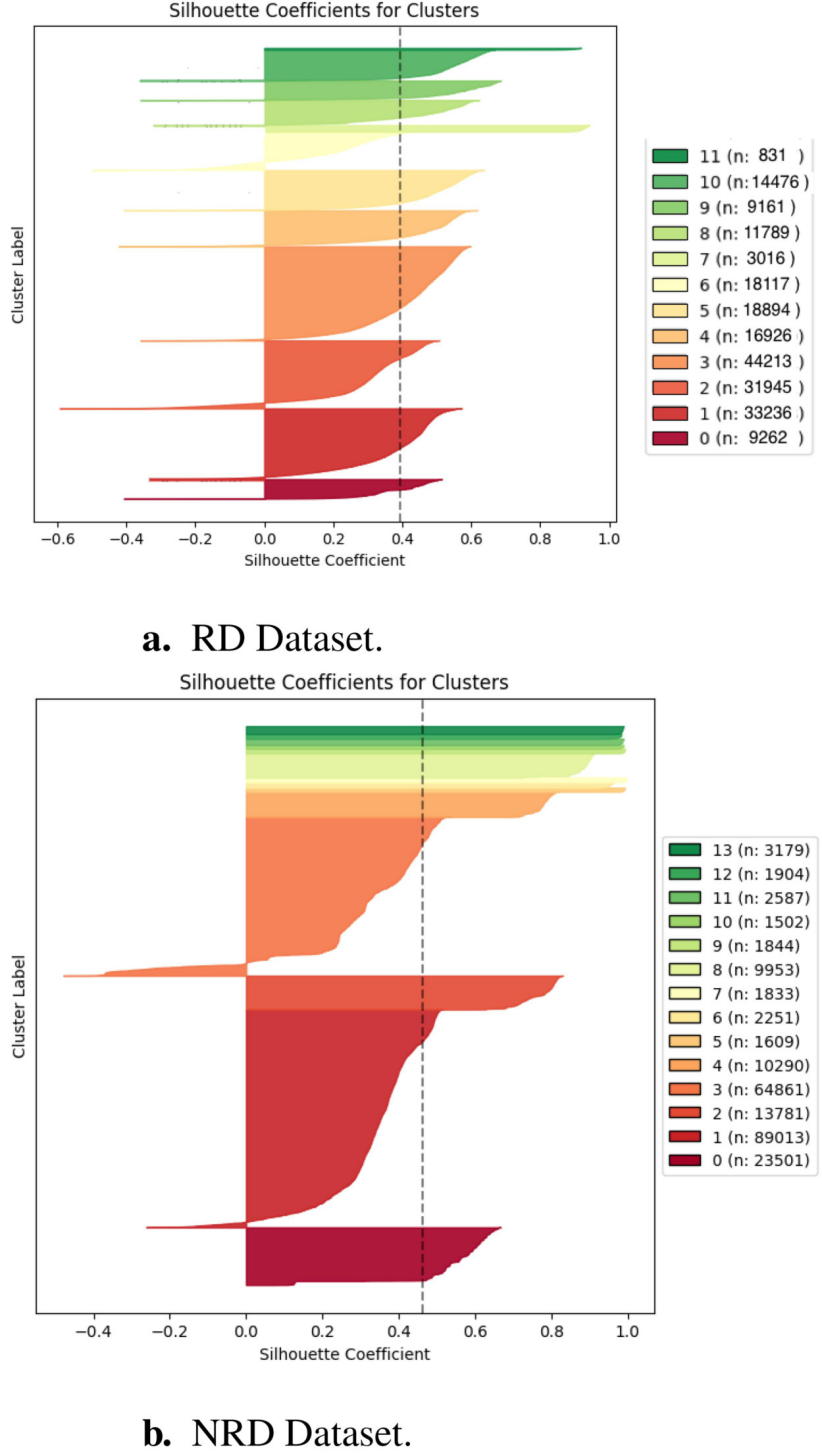
Silhouette plot of the resultant clusters for the RD **(a)** and NRD **(b)** datasets, showing the silhouette score for each sample and the number of samples in each cluster. The dashed line represents the overall average silhouette score.

Analysis was performed on the resultant 12 clusters from the clustering model chosen using average silhouette score. Figure 2a shows the individual silhouette scores for each sample in the RD category. The silhouette scores for all the clusters are within an acceptable range; however, there is considerable variation. Clusters 2 and 6 see a larger number of negative values, suggesting a greater number of wrongly classified samples. Clusters 11, 7 and 3, however, are much more clearly defined; clusters 11 and 7 show near-perfect silhouette scores and near-zero misclassification. Cluster 3, despite being the largest cluster, has a very small number of misclassified samples. This information should be kept in mind during the individual cluster analysis phase, as better-defined clusters may be more clinically relevant. The spread of values is also acceptable, with cluster sizes being reasonably balanced—the initial problem of a large, dominant cluster was successfully overcome using UMAP dimensionality reduction.

#### 3.3.2 No rare disease model

Similarly, analysis was performed on the resultant 14 clusters from the clustering model chosen using average silhouette score. Figure 2b shows the individual silhouette scores for each sample in the NRD category. The first observation is that these clusters for the NRD cohort are much less uniform. There is significantly more variation in the size of clusters, with cluster 1 being particularly large (*n* = 89,013). This indicates that the patterns of comorbidity amongst patients without a RD may be less consistent than for those with a RD. Again, the silhouette scores for all the clusters are within an acceptable range. Whilst the average silhouette score for the NRD category is not significantly higher, there are considerably fewer incorrectly classified records. Only clusters 1 and 3 show incorrectly classified records, with all other records having a positive silhouette score. There are also considerably more clusters with a near-perfect silhouette score, with clusters 5 through 13 all having a silhouette score >0.8. This indicates that the NRD category has a number of very well-defined patterns; although they make up a minority of the dataset. These details should be kept in mind when considering the following clustering analysis section—clusters with a better silhouette score are likely to be more clinically relevant.

### 3.4 Cluster analysis—rare disease dataset

In this section we report the patterns identified for each cluster obtained from the RD sub-dataset. For each of the clusters, the diseases with the greatest positive prevalence difference are shown in a table, alongside details on the cluster’s sex and age characteristics. Table 4 contains demographic information broken down by cluster for both the RD and NRD groups.

**Table 4:** Demographic breakdown by cluster.

Cluster (RD)	Mean age	% Male,% Female	Mean BMI	% White	% Black/Black British	% Asian/Asian British	% Other
Cluster 0	57	46.5, 54.5	26.49	99.7	–	–	0.3
Cluster 1	57	51.2, 48.8	28.53	93.6	1.8	2.8	1.8
Cluster 2	56	47.2, 52.8	28.69	95.5	1.3	1.7	0.5
Cluster 3	57	66.2, 33.8	29.35	93.8	1.4	3.3	1.5
Cluster 4	57	35.2, 64.8	27.16	94.4	1.7	2.0	1.9
Cluster 5	56	40.2, 59.8	27.12	95.1	1.5	1.8	1.6
Cluster 6	57	36.6, 63.4	27.73	96.1	1.3	1.2	1.0
Cluster 7	41	0.2, 99.8	26.13	89.6	3.5	3.0	3.9
Cluster 8	60	87.4, 12.6	27.19	96.2	1.7	1.0	1.1
Cluster 9	55	26.9, 73.1	27.08	94.5	1.9	1.3	2.3
Cluster 10	59	44.4, 55.6	27.33	96.8	1.0	–	2.2
Cluster 11	51	41.6, 58.4	27.29	97.0	1.1	–	1.9
Cluster (NRD)							
Cluster 0	54	49.2, 50.8	26.70	95.0	1.5	1.7	1.8
Cluster 1	57	44.9, 55.1	27.88	94.9	1.7	1.8	1.6
Cluster 2	54	0.1, 99.9	25.96	95.9	1.0	1.3	1.7
Cluster 3	55	50.1, 49.9	26.65	94.6	1.6	1.8	2.0
Cluster 4	57	32.5, 67.5	26.70	97.2	0.6	1.1	1.1
Cluster 5	61	36.3, 63.7	26.42	93.3	2.1	2.8	1.8
Cluster 6	53	73.3, 26.7	26.89	94.2	2.1	1.6	2.1
Cluster 7	53	47.0, 53.0	26.56	93.9	2.1	1.6	2.3
Cluster 8	44	0.0, 100.0	25.47	90.7	3.0	2.7	3.6
Cluster 9	54	44.5, 55.5	26.33	95.3	1.3	1.5	1.9
Cluster 10	47	72.0, 28.0	25.97	92.3	2.3	2.7	2.7
Cluster 11	53	52.4, 47.6	26.45	96.4	1.4	1.2	1.0
Cluster 12	54	37.0, 63.0	26.50	92.6	3.1	1.4	2.9
Cluster 13	54	0.0, 100.0	26.12	91.1	28	2.9	3.6

#### 3.4.1 Cluster 0: melanoma & ophthalmic disorders (*n* = 9,262)

This cluster is characterised by a high prevalence (≈ 63.5%) of “Melanoma and other malignant neoplasms of the skin,” accompanied by high prevalence of numerous ophthalmic diseases (Table 5). The age and sex distribution of this cluster follow those of the wider Biobank dataset. Cluster ethnicity: 99.7% White, 0.3% other. Mean BMI: 26.49.

**Table 5:** Results for Cluster 0: melanoma & ophthalmic disorders in the RD sub-dataset.

Disease category	Prevalence in cluster	Prevalence in RD dataset
	(%, 3 s.f.)	(%, 3 s.f.)
Melanoma and other malignant neoplasms of skin	63.5	10.2
Disorders of lens	46.3	18.0
Disorders of sclera, cornea, iris and ciliary body	19.7	2.51
Disorders of choroid and retina	23.2	6.62
Disorders of ocular muscles, binocular movement, accommodation and refraction	20.1	3.61

#### 3.4.2 Cluster 1: metabolic disorders (*n* = 33,236)

This cluster is characterised by high prevalence of metabolic disorders (≈ 96.6%) and hypertensive diseases (≈ 66.4%) (Table 6). Other diseases do not show a significant increase in prevalence in this cluster when compared to the wider RD sub-dataset—there is a slight increased prevalence of Arthrosis and Diabetes Mellitus. The age distribution for this cluster follows that of the wider Biobank dataset. There is a slight majority of males (51.2%, *n* = 17,031 males vs. *n* = 16,205 females). Cluster ethnicity: 93.6% White, 2.8% Asian/Asian British, 1.8% Black/Black British, 1.8% other. Mean BMI: 28.53.

**Table 6:** Results for Cluster 1: metabolic disorders in the RD sub-dataset.

Disease category	Prevalence in cluster	Prevalence in RD dataset
	(%, 3 s.f.)	(%, 3 s.f.)
Metabolic disorders	96.6	44.7
Hypertensive diseases	66.4	49.5

#### 3.4.3 Cluster 2: gastrointestinal & joint diseases (*n* = 31,945)

This cluster is characterised by high prevalence of Gastrointestinal disorders and joint diseases (Table 7). The age and sex distributions of this cluster follow those of the wider UK Biobank dataset. Cluster ethnicity: 95.5% White, 1.7% Asian/Asian British, 1.3% Black/Black British, 0.5% other. Mean BMI: 28.69.

**Table 7:** Results for Cluster 2: gastrointestinal & joint diseases in the RD sub-dataset.

Disease category	Prevalence in cluster	Prevalence in RD dataset
	(%, 3 s.f.)	(%, 3 s.f.)
Hernia	85.7	26.4
Diseases of oesophagus, stomach and duodenum	94.1	37.8
Symptoms and signs involving the digestive system and abdomen	51.3	32.9
Other diseases of intestines	59.1	40.9
Arthrosis	39.8	27.6
Other joint disorders	24.2	16.7

#### 3.4.4 Cluster 3: cardiovascular, respiratory & metabolic disorders (incl. Diabetes) (*n* = 44,213)

This cluster is characterised by high prevalence of cardiovascular and respiratory disorders (Table 8). This cluster also contains the highest prevalence of Diabetes Mellitus compared with other clusters. The age distribution for this cluster follows that of the wider UK Biobank dataset. However, there is a large majority of males (66.2%, *n* = 29,219 males vs. *n* = 14,994 females). Cluster ethnicity: 93.8% White, 3.3% Asian/Asian British, 1.4% Black/Black British, 1.5% other. Mean BMI: 29.35.

**Table 8:** Results for Cluster 3: cardiovascular, respiratory & metabolic disorders (incl. Diabetes) in the RD sub-dataset.

Disease category	Prevalence in cluster	Prevalence in RD dataset
	(%, 3 s.f.)	(%, 3 s.f.)
Ischaemic heart diseases	83.0	21.5
Metabolic disorders	86.5	44.7
Hypertensive diseases	85.0	49.5
Other forms of heart disease	64.3	25.2
Symptoms and signs involving the circulatory and respiratory systems	61.3	30.9
Renal failure	32.6	14.4
Diabetes mellitus	31.0	15.4

#### 3.4.5 Cluster 4: diseases of oesophagus, stomach and duodenum in younger females (*n* = 16,926)

This cluster is characterised by the high prevalence of “Diseases of oesophagus, stomach and duodenum,” particularly amongst females (Table 9). The age distribution has followed the same shape as the wider UK Biobank dataset; however it is skewed slightly younger (mean 57 years old). This cluster has a large majority of females (64.8%, *n* = 10,962 females vs. *n* = 5,964 males). Cluster ethnicity: 94.4% White, 2.0% Asian/Asian British, 1.7% Black/Black British, 1.9% other. Mean BMI: 27.16.

**Table 9:** Results for Cluster 4: diseases of oesophagus, stomach and duodenum in younger females in the RD sub-dataset.

Disease category	Prevalence in cluster	Prevalence in RD dataset
	(%, 3 s.f.)	(%, 3 s.f.)
Diseases of oesophagus, stomach and duodenum	98.6	37.8
Symptoms and signs involving the digestive system and abdomen	46.7	33.0
Other diseases of intestines	46.1	40.9
Noninflammatory disorders of female genital tract	21.3	15.8

#### 3.4.6 Cluster 5: low comorbidity (*n* = 18,894)

The prevalence of diseases within this cluster is approximately equivalent (within 3%) to the prevalence of diseases in the RD dataset as a whole. There is no distinguishing pattern of comorbidity within this cluster. The mean age within the cluster is 56, and it shows a female majority (59.8%, *n* = 11,300 females vs. *n* = 7,594 males). Cluster ethnicity: 95.1% White, 1.8% Asian/Asian British, 1.5% Black/Black British, 1.6% other. Mean BMI: 27.12.

#### 3.4.7 Cluster 6: other diseases of intestines & joint disorders with benign neoplasms (*n* = 18,117)

This cluster shows a comorbidity pattern reminiscent of Cluster 2, but with a marked prevalence of “Benign Neoplasms” and a greater female majority (Table 10). The mean age within the cluster is 57, and there is a considerable female majority (63.4%, *n* = 11,301 females vs. *n* = 6,816 males). Cluster ethnicity: 96.1% White, 1.2% Asian/Asian British, 1.3% Black/Black British, 1% other. Mean BMI: 27.73.

**Table 10:** Results for Cluster 6: other diseases of intestines & joint disorders with Benign Neoplasms in the RD sub-dataset.

Disease category	Prevalence in cluster	Prevalence in RD dataset
	(%, 3 s.f.)	(%, 3 s.f.)
Other diseases of intestines	77.3	40.9
Benign neoplasms	41.9	24.3
Arthrosis	35.7	27.7
Other joint disorders	26.5	16.7

#### 3.4.8 Cluster 7: female reproductive & birth conditions (*n* = 3,016)

This cluster is made up almost entirely of females (>99%), and has high prevalence of several female reproductive issues (Table 11). This cluster has the lowest mean age at 41. A vast majority of the patients in this cluster lie in the 40–50 age range, with minimal patients in the 50-55 range and the oldest patient in the 55–60 range. Cluster ethnicity: 89.6% White, 3.0% Asian/Asian British, 3.5% Black/Black British, 3.9% other. Mean BMI: 26.13.

**Table 11:** Results for Cluster 7: female reproductive & birth conditions in the RD sub-dataset.

Disease category	Prevalence in cluster	Prevalence in RD dataset
	(%, 3 s.f.)	(%, 3 s.f.)
Persons encountering health services in circumstances related to reproduction	99.7	3.64
Complications of labour and delivery	88.6	1.58
Maternal care related to the fetus and amniotic cavity and possible delivery problems	69.6	1.24
Noninflammatory disorders of female genital tract	36.7	15.8
Benign neoplasms	27.6	24.3
Disorders of breast	5.24	2.29

#### 3.4.9 Cluster 8: urinary & male genital disorders (*n* = 11,789)

This cluster is characterised by high prevalence of conditions relating to the urinary system and male genital organs (Table 12). The mean age within this cluster is 60, with a large majority of males (87.4%, *n* = 10,304 manes vs. females *n* = 1,485 females). Cluster ethnicity: 96.2% White, 1.0% Asian/Asian British, 1.7% Black/Black British, 1.1% other. Mean BMI: 27.19.

**Table 12:** Results for Cluster 8: urinary & male genital disorders in the RD sub-dataset.

Disease category	Prevalence in cluster	Prevalence in RD dataset
	(%, 3 s.f.)	(%, 3 s.f.)
Malignant neoplasms of male genital organs	55.2	6.72
Other diseases of urinary system	57.3	19.6
Symptoms and signs involving the urinary system	53.6	18.2
Diseases of male genital organs	45.3	12.7
Persons encountering health services for specific procedures and health care	49.3	9.64
Abnormal findings on examination of blood, without diagnosis	15.8	8.15
Malignant neoplasms of urinary tract	8.24	2.67

#### 3.4.10 Cluster 9: benign neoplasia (*n* = 9,161)

This cluster is characterised by a high prevalence of benign neoplasms, in combination with disorders associated with the female genital tract and pelvic organs (Table 13). The mean age of this cluster is younger, at 55 years old. There is a majority of females (73.1%, *n* = 6,699 females vs. *n* = 2,462 males). Cluster ethnicity: 94.5% White, 1.3% Asian/Asian British, 1.9% Black/Black British, 2.3% other. Mean BMI: 27.08.

**Table 13:** Results for Cluster 9: benign neoplasia in the RD sub-dataset.

Disease category	Prevalence in cluster	Prevalence in RD dataset
	(%, 3 s.f.)	(%, 3 s.f.)
Benign neoplasms	99.3	24.3
Noninflammatory disorders of female genital tract	33.3	15.8
Melanoma and other malignant neoplasms of skin	16.8	10.3
Inflammatory diseases of female pelvic organs	7.14	2.45
Malignant neoplasms of female genital organs	4.82	2.61

#### 3.4.11 Cluster 10: malignant neoplasia & mixed diseases (14,476)

This cluster is characterised by high prevalence of malignant neoplasms of various body systems, accompanied by a high prevalence of other diseases in various body systems (Table 14). The mean age of patients in this cluster is 59. It has a slight female majority (55.6%, *n* = 8,048 females vs. *n* = 6,428 males). Cluster ethnicity: 96.8% White, 1.0% Black/Black British, 2.2% other. Mean BMI: 27.33.

**Table 14:** Results for Cluster 10: malignant neoplasia & mixed diseases in the RD sub-dataset.

Disease category	Prevalence in cluster	Prevalence in RD dataset
	(%, 3 s.f.)	(%, 3 s.f.)
Malignant neoplasms of ill-defined, secondary and unspecified sites	73.9	9.65
Persons encountering health services for specific procedures and health care	89.5	39.7
Malignant neoplasms of digestive organs	45.1	6.50
Other diseases of blood and blood-forming organs	30.8	4.86
Other bacterial diseases	29.6	7.33
Drugs, medicaments and biological substances causing adverse effects in therapeutic use	28.1	7.13
General symptoms and signs	52.1	31.9
Malignant neoplasms of respiratory and intrathoracic organs	18.0	2.87

#### 3.4.12 Cluster 11: self-harm, poisoning & mental disorders (*n* = 831)

This cluster is characterised by increased prevalence of “Intentional self-harm,” several mental disorders, and issues caused by drugs and psychoactive substances (Table 15). It also has a comparatively high prevalence of “potential health hazards related to socioeconomic and psychosocial circumstances.” The mean age of this cluster is low, at 51. There is a slight majority of females (58.4%, *n* = 485 females vs. *n* = 346 males). Cluster ethnicity: 97.0% White, 1.1% Black British, 1.9% other. Mean BMI: 27.29.

**Table 15:** Results for Cluster 11: self-harm, poisoning & mental disorders in the RD sub-dataset.

Disease category	Prevalence in cluster	Prevalence in RD dataset
	(%, 3 s.f.)	(%, 3 s.f.)
Intentional self-harm	98.1	1.35
Poisoning by drugs, medicaments and biological substances	98.2	1.94
Mood [affective] disorders	84.7	10.5
Mental and behavioural disorders due to psychoactive substance use	51.0	10.7
Neurotic, stress-related and somatoform disorders	43.7	8.36
Persons with potential health hazards related to socioeconomic and psychosocial circumstances	26.1	3.60
Symptoms and signs involving cognition, perception, emotional state and behaviour	27.1	9.86

### 3.5 Cluster analysis—no rare disease dataset

Clusters attained from the NRD dataset show very different composition to those observed from the RD dataset. Many contain a group of patients that share one diagnosis almost exclusively—a phenomenon that is rarely seen in patients diagnosed with a RD.

#### 3.5.1 Cluster 0: low incidence A (*n* = 23,501)

Records in Cluster 0 show no real distinguishable diagnosis pattern, and have a very low rate of diagnosis—even the most prevalent condition in the cluster (“Diseases of oral cavity, salivary glands and jaws”) has only ≈ 7.79% prevalence. The mean age in the cluster is 54, and the sexes are very balanced (50.8% female (*n* = 11,946 female vs. *n* = 11,555 male)). Cluster ethnicity: 95.0% White, 1.7% Asian/Asian British, 1.5% Black/Black British, 1.8% other. Mean BMI: 26.70.

#### 3.5.2 Cluster 1: hypertension, gastrointestinal diseases & joint disorders (*n* = 89,013)

This cluster is characterised by high prevalence of hypertension, in combination with gastrointestinal diseases and joint disorders (Table 16). It is similar in composition to Clusters 6 and 2 observed in the RD cohort. The mean age of patients in this cluster is 57, and there is a majority of females (55.1%, *n* = 49,071 females vs. *n* = 39,942 males). Cluster ethnicity: 94.9% White, 1.8% Asian/Asian British, 1.7% Black/Black British, 1.6% other. Mean BMI: 27.88.

**Table 16:** Results for Cluster 1: hypertension, gastrointestinal diseases & joint disorders in the NRD sub-dataset.

Disease category	Prevalence in cluster	Prevalence in NRD dataset
	(%, 3 s.f.)	(%, 3 s.f.)
Hypertensive diseases	48.6	20.3
Other diseases of intestines	49.6	22.0
Arthrosis	27.3	15.1
Diseases of oesophagus, stomach and duodenum	25.3	14.8
Hernia	21.7	12.7
Other joint disorders	19.8	11.5
Symptoms and signs involving the digestive system and abdomen	24.4	16.9

#### 3.5.3 Cluster 2: noninflammatory disorders of female genital tract, & benign neoplasms (*n* = 13,781)

Almost every patient in this cluster was diagnosed with “Noninflammatory disorders of female genital tract” which has a prevalence ≈ 99.98%. There was also an increased prevalence of “Benign neoplasms,” with a prevalence ≈ 28.0%, against a full dataset prevalence ≈ 12.4%. Other diagnoses in this cluster were very low-incidence (<10%). The mean age of patients in this cluster is 54, and >99% patients are female. Cluster ethnicity: 95.9% White, 1.3% Asian/Asian British, 1.0% Black/Black British, 1.7% other. Mean BMI: 25.96.

#### 3.5.4 Cluster 3: low incidence B (*n* = 64,861)

This cluster, like Cluster 0, has few distinguishable patterns. It must be noted that this cluster was the most poorly defined of the results, and hence could likely be split differently with a change in methodology. The only conditions that showed an increased prevalence over that of the NRD dataset were “Symptoms and signs involving the circulatory and respiratory systems,” “General symptoms and signs,” and “Persons encountering health services for examination and investigation.” These diagnoses are in of themselves very general. The mean age of patients in this cluster is 55, and the sexes are very balanced (50.1% males, *n* = 32,467 males vs. *n* = 32,394 females). Cluster ethnicity: 94.6% White, 1.8% Asian/Asian British, 1.6% Black/Black British, 2.0% other. Mean BMI: 26.65.

#### 3.5.5 Cluster 4: falls & associated injuries (*n* = 10,290)

Almost every patient in this cluster experienced a fall, with a prevalence ≈99.96%. All other prevalent diagnoses are associated with injuries to the limbs and head. In order of prevalence, injuries to: elbow and forearm (≈37.8%), knee and lower leg (≈27.6%), head (≈23.9%), hip and thigh (≈11.5%), shoulder and upper arm (≈10.4%), wrist and hand (≈10.2%). The mean age of patients in this cluster is 57, and there is a large majority of females (67.5%, *n* = 6,950 females vs. *n* = 3,340 males). Cluster ethnicity: 97.2% White, 1.1% Asian/Asian British, 0.6% Black/Black British, 1.1% other. Mean BMI: 26.70.

#### 3.5.6 Cluster 5: disorders of lens (*n* = 1,609)

A vast majority of patients in this cluster have an exclusive diagnosis of “Disorders of lens” (prevalence ≈ 90.3%). All other diagnoses have a prevalence <5%. The mean age of patients in this cluster is 61, and there is a considerable female majority (63.7%, *n* = 1,025 females vs. *n* = 584 males). Cluster ethnicity: 93.3% White, 2.8% Asian/Asian British, 2.1% Black/Black British, 1.8% other. Mean BMI: 26.42.

#### 3.5.7 Cluster 6: exposure to inanimate mechanical forces & associated injuries to the wrist and hand (*n* = 2,251)

This cluster is dominated by high prevalence of “Exposure to inanimate mechanical forces” (≈ 97.9%) and “Injuries to the wrist and hand” (≈ 97.2)%. All other diagnoses show no increase in prevalence over that of the wider NRD dataset. The mean age of patients in his cluster is 53, and there is a large male majority (73.3%, *n* = 1,650 males vs. *n* = 601 females).

#### 3.5.8 Cluster 7: low prevalence C (*n* = 1,833)

This cluster has the lowest rate of diagnosis of all other clusters, and shows no distinguishable patterns. The mean age of patients in this cluster is 53, and the sexes are relatively balanced (53% female, *n* = 971 females vs. *n* = 862 males). Cluster ethnicity: 93.9% White, 1.6% Asian/Asian British, 2.1% Black/Black British, 2.3% other. Mean BMI: 26.56.

#### 3.5.9 Cluster 8: female reproductive & birth conditions (*n* = 9,953)

This cluster shares patterns observed in Cluster 7 of the RD dataset (Table 17). It is made up entirely of females, and has high prevalence of numerous conditions associated with the birth process. The mean age of patients in this cluster is 44, with a vast majority of patients in the 40–45 range. Cluster ethnicity: 90.7% White, 2.7% Asian/Asian British, 3.0% Black/Black British, 1.4% Mixed, 2.2% other. Mean BMI: 25.47.

**Table 17:** Results for Cluster 8: female reproductive & birth conditions in the NRD sub-dataset.

Disease category	Prevalence in cluster	Prevalence in NRD dataset
	(%, 3 s.f.)	(%, 3 s.f.)
Persons encountering health services in circumstances related to reproduction	99.0	7.83
Complications of labour and delivery	81.8	3.71
Maternal care related to the fetus and amniotic cavity and possible delivery problems	55.4	2.57
Diseases of oesophagus, stomach and duodenum	25.3	14.8
Other maternal disorders predominantly related to pregnancy	22.2	1.15
Delivery	21.1	1.11
Pregnancy with abortive outcome	15.6	1.47
Noninflammatory disorders of female genital tract	20.4	15.2

#### 3.5.10 Cluster 9: diseases of veins, lymphatic vessels and lymph nodes, not elsewhere classified (*n* = 1,844)

This cluster is characterised by high prevalence of “Diseases of veins, lymphatic vessels and lymph nodes, not elsewhere classified” (≈ 89.7%) as an exclusive diagnosis. All other diagnoses in this cluster have 5% prevalence. The mean age of patients in this cluster is 54, and there is a slight majority of females (55.5%, *n* = 1,024 females vs. *n* = 820 males). Cluster ethnicity: 95.3% White, 1.5% Asian/Asian British, 1.3% Black/Black British, 1.9% other. Mean BMI: 26.33.

#### 3.5.11 Cluster 10: reproductive health services (*n* = 1,502)

This cluster is characterised by high prevalence of “Persons encountering health services in circumstances related to reproduction” (≈ 92.2%), with no other highly prevalent diagnoses. All other diagnoses have a prevalence <5% in this cluster. The mean age of patients in this cluster is 47, and it has a significant majority of males (72%, *n* = 1,081 males vs. *n* = 421 females). Cluster ethnicity: 92.3% White, 2.7% Asian/Asian British, 2.3% Black/Black British, 2.7% other. Mean BMI: 25.97.

#### 3.5.12 Cluster 11: other diseases of intestines (*n* = 2,587)

This cluster is characterised by a relatively high prevalence of “Other Diseases of the intestine” (≈ 78.0%), with no other prevalent diagnoses. All other diagnoses have a prevalence <5% in this cluster. Its separation of this particular diagnosis from other gastrointestinal conditions is reminiscent of the pattern observed in Cluster 6 of the RD category; however, there is a marked absence of benign neoplasms and joint disorders here. The mean age of patients in this cluster is 53, the sexes are relatively balanced (52.4% males, *n* = 1,355 males vs. *n* = 1,232 females). Cluster ethnicity: 96.4% White, 1.2% Asian/Asian British, 1.4% Black/Black British, 1.0% other. Mean BMI: 26.45.

#### 3.5.13 Cluster 12: benign neoplasms (*n* = 1,904)

This cluster is characterised by high prevalence of benign neoplasms (≈87.6%), with no other prevalent diagnoses. All other diagnoses have a prevalence <5% in this cluster. The separation of this diagnosis from malignant neoplasia is reminiscent of the pattern observed in Cluster 9 of the RD category; however, there is not a high prevalence of disorders associated with the female genital or pelvic organs. The mean age of patients in this cluster is 54, and there is a significant female majority (63%, *n* = 1,200 females vs. *n* = 704 males). Cluster ethnicity: 92.6% White, 1.4% Asian/Asian British, 3.1% Black/Black British, 1.2% Mixed, 1.7% other. Mean BMI: 26.50.

#### 3.5.14 Cluster 13: noninflammatory disorders of the female genital tract (*n* = 3,179)

This cluster consists entirely of females, and sees a very high prevalence of “Noninflammatory disorders of female genital tract” (≈84.8%), with no other highly prevalent diagnoses. All other diagnoses have a prevalence <5% in this cluster. The mean age of patients in this cluster is 54.

### 3.6 Comparison with relative risk and phi coefficient

The most prevalent disease categories within each cluster extracted from the RD group were selected for analysis using Relative Risk (RR) and Phi Coefficient (PHI). Calculated pairwise and across the entire RD dataset, each combination of disease categories was analysed for both directions of RR. The full results of these analyses are available as Supplementary Tables S5–S15. With the exception of Melanoma diagnoses prevalent in Clusters 0 (Supplementary Table S5) and 9 (Supplementary Table S13), RR scores were in agreement with our groupings (RR > 1) and were statistically significant with a 95% confidence interval. PHI results are also in broad agreement regarding the link between disease categories identified in each cluster using this methodology, with all other PHI values being >0.

## 4 Discussion

All of the clusters obtained for the RD category had complex combinations of diagnoses; there was no cluster wherein a highly prevalent diagnosis was not accompanied by at least one other notably prevalent diagnosis. This even held true for clusters that had a diagnosis with >95% prevalence; there were always many diagnoses with a high prevalence. The same cannot be said, however, for the majority of clusters obtained from the NRD group. In fact, half of the clusters obtained for NRDs (5, 6, 9–13) had very high prevalence of one or two diagnoses, and an almost total lack of consistent comorbidities alongside them. This may indicate that, generally, those without a RD do not have as high a level of consistent comorbidity when compared with those who do. A further difference between RD and NRD categories is the distinct lack of “low incidence” clusters amongst the RD cohort. These “low incidence” clusters, three of which are present in the NRD category, consist of patients for which no comorbidity pattern could be identified by the clustering model.

There are a few clusters obtained from both NRD and RD categories that display similar patterns. For instance, both contain a cluster with high prevalence of gastrointestinal diseases and joint disorders; although there is a high prevalence of hypertension in the NRD cluster that is absent from the RD clusters. A cluster characterised by female reproductive and birth conditions is also present in each sub-dataset, with very similar prevalence of diagnoses save for a marked presence of benign neoplasms in the RD category. There was a separation in both cohorts of “Other diseases of the intestines” from other gastrointestinal conditions. Finally, both categories see a cluster of patients characterised by presence of benign neoplasms. Whilst there were few other consistent diagnoses alongside this in the NRD sub-dataset, there was a high prevalence of issues associated with the female genital and pelvic organs in the RD sub-dataset.

Both RD and NRD sub-datasets produced well-defined clusters that were unique to that specific category. The RD category produced three well-defined clusters not seen in the NRD category. These were Cluster 8: “Urinary & Male Genital Disorders,” Cluster 10: “Malignant Neoplasia & Mixed Diseases,” and Cluster 11: “Self-Harm, Poisoning & Mental Disorders.” Cluster 8 and Cluster 11 are of particular clinical interest; the most prevalent rare diagnosis in Cluster 8 is C67.9: Non-papillary transitional cell carcinoma of the bladder, classified by Orphanet as a “rare neoplastic disease.” This is in line with the highlighted comorbidities, that are associated with diseases of the male genital tract and diseases of the urinary system. The most prevalent rare diagnosis in Cluster 11 is T40.2: Acute opioid intoxication, classified by Orphanet as a “rare disorder due to toxic effects.” This is also in line with the highlighted comorbidities, which are associated with poisoning; however it should be noted that the rare disease here is a result of opioid poisoning, not necessarily the cause of the other comorbid conditions. The next most prevalent is E83.4: Disorder of magnesium transport, classified by Orphanet as a “rare inborn error of metabolism.” The link between magnesium and mental health, however, is inconclusive [44], and further work is required to associate specific rare conditions with their comorbidities. None of the patterns identified in these clusters were picked up by the clustering model in the NRD sub-dataset, indicating that they may not be experienced as frequently by those patients. The cluster highlighting “Self-Harm, Poisoning & Mental Disorders” in RD, that was not enriched in NRD, reflects work we have carried out previously in the UK Biobank, revealing a higher burden of anxiety and depression in those living with a RD compared to those with a NRD, or those with no diagnoses. England, Wales, Scotland and Northern Ireland Rare Disease Action Plans all outline the need for improved mental health services for RD patients this as a future focus [45–48]. RDs have been shown to negatively impact mental health [49, 50], with a 2021 systematic review of affective and anxiety disorders in RD highlighting, for example, 44.2% of individuals with a RD suffered anxiety over their lifetime [51].

The NRD category produced five well-defined clusters not seen in the RD category. Excluding the low-incidence clusters, these are Cluster 4: “Falls & Associated Injuries,” Cluster 5: “Disorders of Lens,” Cluster 6: “Exposure to Inanimate Mechanical Forces & Associated Injuries to the Wrist and Hand,” Cluster 9: “Diseases of veins, lymphatic vessels and lymph nodes, not elsewhere classified,” and Cluster 13: “Noninflammatory Disorders of the Female Genital Tract.” Perhaps the most interesting difference here is the lack of clusters associated with accidents in the RD category. Diagnoses such as “Fall” and “Exposure to inanimate mechanical forces” were not prevalent in any RD clusters. Families of individuals with a rare disease experience an increased caregiver burden [52]; it would be interesting to explore if this reduction in falls in RD is due to increased family or caregiver support. The other clusters absent from the RD cohort fall into the previously mentioned category of clusters characterised by near-exclusive prevalence of one diagnosis.

The broad agreement of RR and PHI values for each of the disease category pairs identified within rare disease clusters lends credibility to the results obtained using the clustering algorithm. The advantage attained through use of clustering vs. RR or PHI is that clustering offers a higher-level, multivariate view of comorbidity patterns. As RR and PHI consider conditions pairwise, it can be difficult to identify complex comorbidity patterns consisting of more than two conditions; particularly with a large number of features. As pairwise metrics are strictly dyadic, they may miss higher-order patterns [53]. Clustering addresses this by offering patient-centered analysis of patterns consisting of many conditions across the entire patient cohort, capturing a broader range of comorbidity patterns than when considering isolated disease pairs. Pairwise methods also risk uncovering indirect or superficial associations [54]. Identification of comorbidity patterns at the patient level, rather than the disease level, overcomes these issues through a multivariate approach. Moreover, frequency-based detection (e.g., identifying the most common co-occurring diseases across the full cohort) tends to ignore patient heterogeneity, especially in large-scale datasets like UK Biobank. The method used in this study explicitly preserves and leverages patient heterogeneity, enabling the discovery of cluster-specific comorbidity patterns that may be relevant to subpopulations or underlying pathophysiological mechanisms. Traditional methods may miss these patterns, as they are sensitive to prevalence and sample size [55]. Frequency analysis or itemset mining identifies only the globally most common co-occurring conditions, which risks overlooking rare but clinically meaningful substructures. In contrast, clustering partitions patients into groups with shared multimorbidity profiles, allowing us to detect patterns that emerge only within subsets of the population. This could lead to an interesting area of future work, wherein supervised machine learning models may be trained to diagnose or differentiate a RD based on a patient’s comorbidity profile. The distinct groupings identified in this study suggest that such distinctions exist in the data, and hence future work may harness these patterns in a supervised manner. This would further support the hypothesis of distinct differences in comorbidity profiles between RD and NRD patients.

This research provides a foundational map of comorbidity burden for those living with rare disease, and those with more common diseases. We appreciate that the clinical implications of these clusters require further exploration. Patterns, such as the association between rare disease and mental health align with previous research. Recent studies have highlighted the significant mental health burden faced by individuals with rare diseases (RD). A 2022 UK survey of more than 1,200 RD patients and 500 carers reported substantial negative impacts on mental wellbeing [49]. Similarly, a 2021 systematic review across 24 rare diseases found a lifetime anxiety prevalence of 44.1%, aligning with broader reports on the lived experience of RD patients in the UK [51]. The England, Wales, Scotland and Northern Ireland Rare Disease Action plans highlight the need for improved mental health support for those living with a RD [45–48]. Additional clusters, such as Cluster 8, specific to RD, “Urinary & Male Genital Disorders,” may reveal novel patterns which warrant future study and a focus of resources to understand the potential molecular underpinnings of such associations, and uncover potential clinical impacts. It is hoped that this research will pave the way towards personalised medicine, whereby knowledge of co-occurring conditions may enable stratification of patients to better understand clinical trajectories, risk profiles or care needs. This may be particularly important for those who have not yet received a disease diagnosis, or those who have been misdiagnosed, where traditional diagnostic pathways are sub-optimal. Further research is required to refine symptom and diagnosis clusters for effective clinical application. Improvements in rare disease coding, greater incorporation of genetic, phenotypic and molecular data, advanced use and maintenance of rare disease registries, integration of longitudinal healthcare data across the health system (primary, secondary, prescription, patient reporting and outcomes), replication of clusters across independent datasets (including in under-represented groups), addition of clinical annotation and future tool development (with clinical and patient involvement in design steps) are all important areas of future work.

As our analysis included diagnoses recorded in hospital inpatient records (both primary and secondary ICD-10 code positions), the patterns observed represent co-occurring conditions more broadly, rather than comorbidities in a strict clinical sense [56]. This approach captures acute injuries and short-term illnesses alongside chronic disease. Moreover, the severity of the initial diagnosis may impact coding for secondary diagnoses [56]. This is demonstrated in the NRD cohort, where Cluster 4 highlights the prominence of falls and injury-related diagnoses, likely reflecting more hospital interactions for acute events in NRD compared to RD individuals. These patterns, whilst not strictly comorbidities, can highlight differences in healthcare interactions between those with and without a rare disease.

Whilst our model did not include age or sex as features for cluster analysis, we observed differences across the identified clusters with respect to sex, suggesting that these factors may contribute meaningfully to co-morbidity stratification. Age and sex have strong impacts in disease presentation and comorbidities [57], and including them in the cluster analysis could lead to grouping of patients based on their demographic variables, rather than their diagnosed conditions (in line with the aim of the analysis). By using a model agnostic to demographic variables, data structures that are not driven by known demographic biases can be identified. Moreover, by analysing age and sex distributions in the identified clusters, this work has highlighted key demographic differences which warrant future investigation. For example, with respect to personalised medicine, this information could be useful for shaping risk models, developing early screening strategies, or tailoring interventions.

Overall, our analysis of comorbidity patterns in RD, and comparison with comorbidity patterns in NRD, reveals significant potential for clustering patients with similar comorbidities to enhance sample sizes for downstream analyses. This helps overcome the limitation of low prevalence of individual rare diseases. Moreover, our approach has highlighted critical points of intervention that could be targeted to improve quality of life for patients living with a RD. For example, improving mental health support could lead to better health outcomes, particularly relevant for the cluster of individuals experiencing self-harm, mental disorders, and issues caused by drugs and psychoactive substances alongside their rare disease. By focusing on these areas, healthcare providers could provide more targeted and effective treatments, ultimately enhancing patient care. Knowledge of unique RD comorbidity patterns improves our knowledge regarding the disease burden experienced by those living with a rare disease, advances scientific research and paves the way for more patient-centered healthcare strategies.

### 4.1 Limitations & future work

The computational approach used in this research allowed the RD and NRD groups to express distinct structure without presupposing comparability. However, a limitation of this method is that demographic confounders may be introduced. Therefore, information relating to age, sex, ethnicity and BMI features across clusters has been shown to highlight these potential disparities, which are potentially informative for downstream research to improve our fundamental understanding of rare disease comorbidities. They may reflect broader structural disparities in health access, diagnosis, and outcomes which warrant future comprehensive investigation. Wider research is needed to identify how demographic influences, health behaviours, and social determinants of health intersect with rare disease burden, which may reveal important dimensions of health inequality and opportunities for more equitable precision medicine strategies.

Hierarchical clustering was the selected algorithm for this study, as the goal was to obtain clear, interpretable groupings that may not be identified by algorithms that assume spherical groupings; particularly when considering complex diagnosis data. Hierarchical clustering has been shown to be effective in numerous previous studies when applied to data of this nature. Cluster coherence was assessed using Silhouette Score, a widely used metric for internal cluster result validation. This is not a definitive metric, as it is not compared against a ground truth—it is used as a supportive tool to assess cluster coherence. Although our results displayed consistent, clinically meaningful patterns within the derived clusters using Hierarchical Clustering, future work may utilise different clustering algorithms to both verify and expand upon results seen in this study.

An interesting area of future research will be to assess if the differences observed in comorbidity patterns are impacted by surveillance bias. For example, individuals with a rare disease may undergo more frequent medical examinations, which could increase the detection of comorbidities. Prior research using phenotype-genotype comorbidity analysis has shown that clustering phenotypes in rare disease may highlight underlying mechanisms of multimorbidity [58]. The present research provides insights into comorbidity sub-clusters which warrant future investigation and validation at a phenotypic, genetic and molecular level. Interesting future work also could focus on temporal patterns of disease onset, which may help understand the rare disease diagnostic journeys and identify significant commodity patterns [59].

As this study focuses on ICD-10 codes derived from hospital inpatient records, the findings reflect differences in inpatient healthcare patterns between individuals with a rare disease and those with only non-rare diseases. Further work is required to determine whether these clusters generalise to non-inpatient healthcare settings. This is particularly relevant for clusters such as Self-harm, poisoning, and mental disorders (Cluster 11, RD), where future investigation is needed to assess whether the more severe mental health presentations observed among individuals with a rare disease are also evident in other areas of healthcare, for example primary care. Our work highlights important differences in how RD and NRD groups engage with hospital services. While these patterns may reflect broader trends, we hope that our findings can guide future research aimed at understanding the lived experiences of people with rare diseases and ultimately inform earlier interventions to improve care and prevent more serious health outcomes.

It is also important to highlight that the healthcare experience for RD patients is different, as they often experience fragmented care, transition challenges and a limited awareness of their disease [46, 60]. This too may impact comorbidity patterns, which are reflective of underlying healthcare inequalities which warrant future investigation. Quality standards are in development for rare disease in the UK to define standards for high-quality and sustainable care, treatment and management of rare disease [61].

Population-level resources such as the UK Biobank can offer valuable insights into rare disease as a whole; however, future work is needed for disease-specific granularity. Notably, only 8% of rare disease have a specific ICD-10 code [62], limiting the ability to explore condition-specific associations. Advancing diagnostic coding and improving rare disease fine mapping will enable more granular insights into comorbidities in specific rare diseases [35]. The absence of fine Orphanet (ORPHA) to ICD-10 mapping in this study may have inflated the apparent size of the rare disease cohort. Multiple independent expert reviewers may conduct a more granular consensus mapping approach by validating each of the ICD-10 code to Orphanet disease pairs, as seen in Patrick et al. [35]. This would produce a more specific classification of RD vs. NRD. As this research focused on broader ICD-10 patterns from hospital records across rare and common diseases, this level of detailed mapping was not undertaken. This represents both a limitation of our work and a wider challenge in rare disease epidemiology that warrants further investigation, and future work should carry out this more intensive mapping to validate results. It also highlights the drawbacks of relying on healthcare-setting ICD-10 data for detailed rare disease research. We recognise that many individuals living with a rare disease remain undiagnosed or do not present with a specific ICD-10 code; therefore, some individuals with a rare disease may have been misclassified into the NRD group in this study. Any misclassification is likely to dilute observable differences between RD and NRD groups, meaning our findings may represent conservative estimates. This is a limitation of the present work and, more broadly, a limitation in rare disease research that requires future work to overcome.

Evidence suggested that the UK Biobank may have “healthy volunteer bias” [63], which could limit the rare disease and non-rare disease groups in this study, and limit diagnoses available for study. This is a limitation of the present study and future work is required to validate findings is wider, more diverse population, in terms of ethnicity, age, socioeconomic status and health.

The clusters identified here reflect associations and not causal relationships. Future work is needed to determine if these relationships are due to shared risk factors, such as genetics or lifetime exposures, including the environment or healthcare utilisation. Longitudinal studies, following diagnostic pathways over time, as well as enhanced molecular and clinical variables, will provide opportunities to determine the directionality and clinical important of such associations.

## Data Availability

The data analyzed in this study is subject to the following licenses/restrictions: the data used in this research is available from UK Biobank subject to standard procedures. Requests to access these datasets should be directed to https://www.ukbiobank.ac.uk/use-our-data/apply-for-access/.
