# Supplementary material for: Detecting comorbidity patterns in rare disease patients with machine learning
Source: Front Epidemiol. 2026 May 4;6:1765678. doi: 10.3389/fepid.2026.1765678 (PMC13180900; doi:10.3389/fepid.2026.1765678)
Supplement: Supplementary file 1 [file Datasheet1.pdf]

Supplementary Material

Model Selection

To select the optimal clustering model, the average silhouette score was analsyed for differing numbers of UMAP dimensions and resultant clusters. The heatmap in Figure S2 (A) displays the silhouette scores obtained for different cluster setups on the RD sub-dataset. The first observation is that whilst the variance in average silhouette scores is clearly observable, it does not have a large range. The minimum average silhouette score attained was ≈ 0.309, and the maximum ≈ 0.394. This indicates that for each of the setups there were meaningful clusters found. The number of dimensions has a reduced effect on the average silhouette score compared to the number of resultant clusters, although higher numbers of dimensions show increasingly poor results.

The hypothesis attributing poor performance to high dimensionality holds - as the data tends back toward its high-dimensional roots, the model performance decreases. The optimal number of UMAP dimensions for the dataset was found to be between 10 and 30. The number of clusters chosen has a more pronounced effect on the average silhouette score. Across dimension numbers, the 12-15 range yields the best results. This is in line with expected results from previous studies in a similar context(1, 2, 3). Fewer clusters than this show greatly reduced performance - it is likely that a small number of clusters is not sufficient to represent the complexities of the large dataset. Minor reductions in performance are shown as the number of clusters increases beyond 12; however, more than 15 clusters would not be clinically manageable and so were not further explored in this study. It was determined that the optimal model, with silhouette score = 0.394, was composed of 30 UMAP dimensions and 12 resultant clusters. Average cluster linkage was used with the Euclidean distance metric.

Figure S2 (B) displays the results for the NRD dataset. Outcomes with number of clusters in the range 10-15 were chosen in order to make comparison with the results from the RD dataset more intuitive. The first observation is that the average silhouette scores are notably higher for this dataset, with a minimum ≈ 0.358 and maximum ≈ 0.463. The explanation for this will become clear in the following 'Cluster Analysis' section, however it is largely due to a lower rate of comorbidity in this dataset; many records consist of only one diagnosis. An increasing number of dimensions shows mildly improving average silhouette scores, converse to the results attained for the NRD dataset. This may be also be attributed to the simpler nature of the NRD dataset, as there are fewer complex relations between datapoints and hence reducing the dimensionality is less effective. The number of outcome clusters has a less pronounced effect here than on the RD dataset, although the pattern of improved average silhouette scores with increasing number of clusters is preserved. This also falls in line with results from previous studies in a similar context. It was determined that the optimal clustering setup, with silhouette score = 0.463, was composed of 50 UMAP dimensions and 14 resultant clusters. Average cluster linkage was used with the Euclidean distance metric.

Methodology

Silhouette Coefficient (SC)

The silhouette coefficient measures how well a single observation fits within its assigned cluster compared to other clusters. For a given sample i: Let a_i be the mean distance from i to all other points in its own cluster. Let b_i be the be the minimum average distance from i to points in any other cluster.

Then silhouette coefficient:

$$s_i = \frac{b_i - a_i}{\max(a_i, b_i)} - 1$$

Such that:

$$-1 \leq s_i \leq 1$$

Interpretation of SC is intuitive. Values close to 1 indicate strong cluster membership, values close to 0 indicate boundary points, and values close to -1 indicate potential misclassifications. In this study, the mean of all SC values is taken to measure the quality of the clustering setup as a whole.

Relative Risk (RR) and Phi Coefficient

Assume a 2×2 contingency table for two binary diagnoses D_1 and D_2:

	$D_2 = 1$	$D_2 = 0$
$D_1 = 1$	a	b
$D_1 = 0$	c	d

Each binary diagnosis field can have a value of either 0 or 1. The values *a, b, c,* and *d* represent the probability of each of the four possible states for two binary diagnosis fields. As RR is asymmetric, it is necessary to calculate it in two directions. The first direction answers the question: "How much more likely is diagnosis D_2 among patients who have diagnosis D_1, compared to patients who do not have D_1?"

Therefore RR direction 1:

$$RR_{D_2|D_1} = \frac{P(D_2 = 1 \mid D_1 = 1)}{P(D_2 = 1 \mid D_1 = 0)}$$

The second direction represents the converse: "How much more likely is diagnosis D_1 among patients who have diagnosis D_2, compared to patients who do not have D_2?"

Therefore RR direction 2:

$$RR_{D_1|D_2} = \frac{P(D_1 = 1 \mid D_2 = 1)}{P(D_1 = 1 \mid D_2 = 0)}$$

Interpretation of RR values is intuitive. If RR direction 1 ($RR_{D_2|D_1}$) >1, then D_2 is more common among patients with D_1. If $RR_{D_2|D_1}$ = 2, then patients with D_1 are twice as likely to also have D_2.

Phi Coefficient is a symmetric measure of association between two binary variables. It captures overall strength of correlation, not risk amplification.

Phi Coefficient:

$$\phi = \frac{ad - bc}{\sqrt{(a+b)(c+d)(a+c)(b+d)}}$$

Such that:

$$-1 \leq \phi \leq 1$$

Interpretation of Phi Coefficient is also intuitive. ϕ >0 indicates diagnoses co-occur more often than expected under independence. ϕ <0 indicates diagnoses co-occur less often than expected under independence. ϕ = 0 indicates no association between variables. ϕ has a maximum value of 1, indicating strong co-occurrence, and a minimum value of -1, indicating strong opposite occurrence.

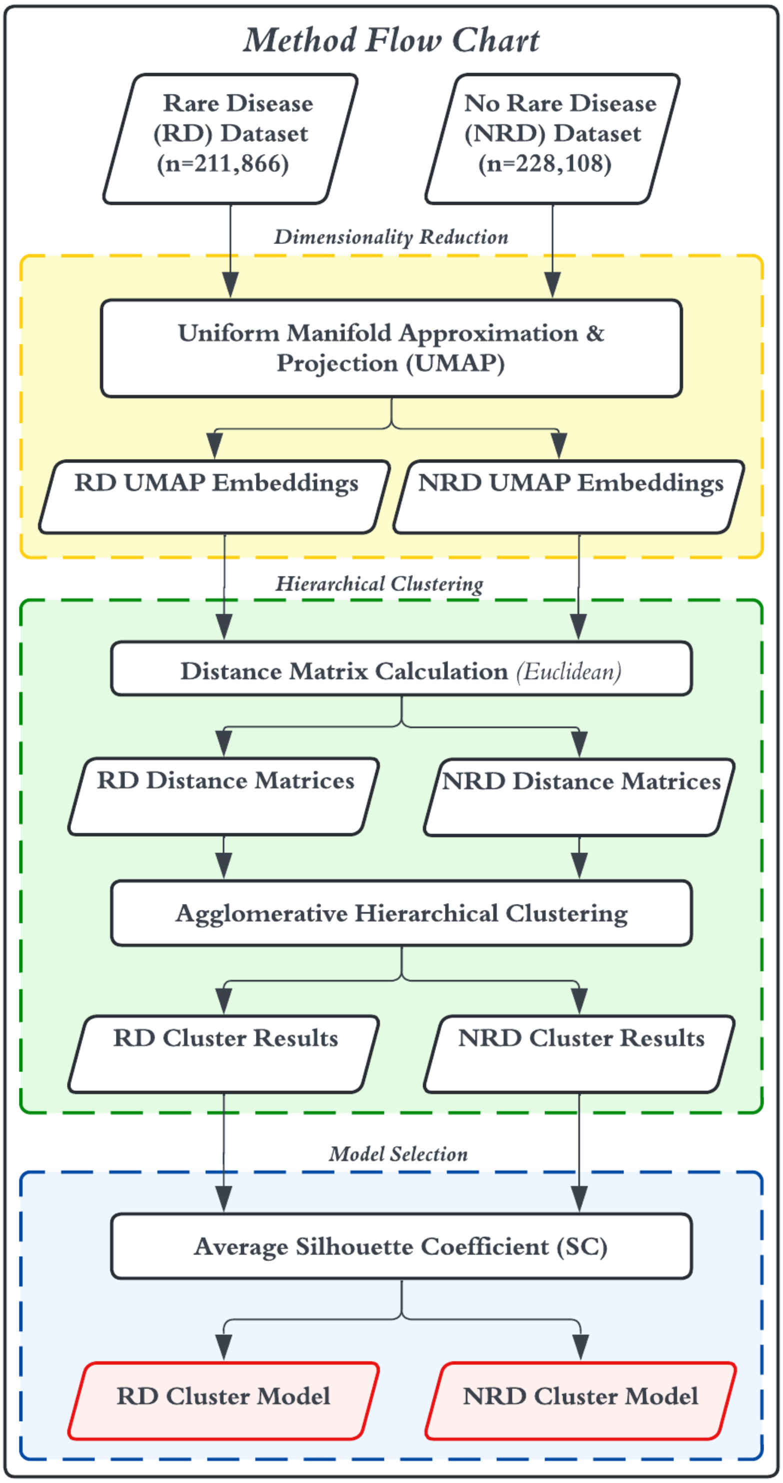

Figure S1. Pipeline of the method implemented for our comorbidity study.

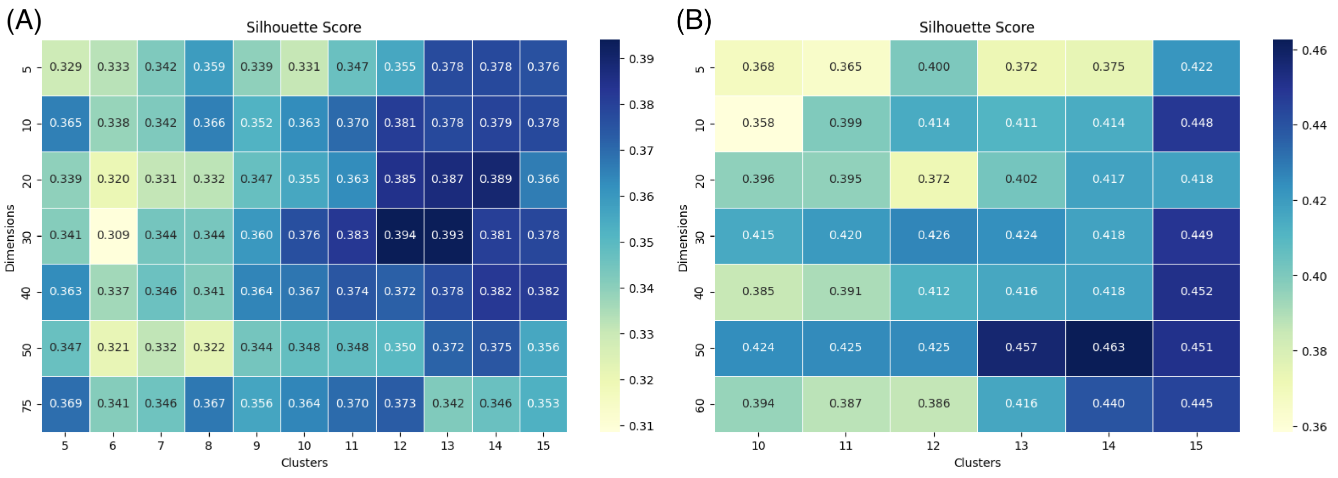

Figure S2. Heatmap of Silhouette Scores for RD **(A)** and NRD **(B)** datasets according to number of dimensions and resultant clusters.

REFERENCES

1. Newcomer SR, Steiner JF, Bayliss EA. Identifying subgroups of complex patients with cluster analysis. The American journal of managed care. 2011 Aug 1;17(8):e324-32.
2. Singh SP, Karkare S, Baswan SM, Singh VP. Agglomerative hierarchical clustering analysis of co/multi-morbidities. arXiv preprint arXiv:1807.04325. 2018 Jul 11.
3. Karkare R. Clustering Analysis to Explore Cohorts in Comorbid Patients. bioRxiv. 2018 Aug 24:396481.

Table S1. Disease Categories and Diagnosis Counts in RD and NRD Groups (Part 1)

ICD-10 Range	Description	Diagnoses in RD	Diagnoses in NRD
A00-A09	Intestinal infectious diseases	19343	6492
A30-A49	Other bacterial diseases	15525	N/A
B00-B09	Viral infections characterized by skin and mucous membrane lesions	2466	N/A
B25-B34	Other viral diseases	2678	N/A
B35-B49	Mycoses	6844	N/A
B95-B98	Bacterial, viral and other infectious agents	24329	6304
C15-C26	Malignant neoplasms of digestive organs	13765	N/A
C30-C39	Malignant neoplasms of respiratory and intrathoracic organs	6078	N/A
C43-C44	Melanoma and other malignant neoplasms of skin	21802	5230
C50-C50	Malignant neoplasm of breast	10153	7703
C51-C58	Malignant neoplasms of female genital organs	4688	N/A
C60-C63	Malignant neoplasms of male genital organs	14227	N/A
C64-C68	Malignant neoplasms of urinary tract	5658	N/A
C76-C80	Malignant neoplasms of ill-defined, secondary and unspecified sites	20446	3089
C81-C96	Malignant neoplasms, stated or presumed to be primary, of lymphoid, haematopoietic and related tissue	6560	N/A
D00-D09	In situ neoplasms	5787	3729
D10-D36	Benign neoplasms	51515	28388
D37-D48	Neoplasms of uncertain or unknown behaviour	8502	N/A
D50-D53	Nutritional anaemias	19600	4301
D60-D64	Aplastic and other anaemias	24407	6132
D65-D69	Coagulation defects, purpura and other haemorrhagic conditions	6118	N/A
D70-D77	Other diseases of blood and blood-forming organs	10290	N/A
E00-E07	Disorders of thyroid gland	22980	11648
E10-E14	Diabetes mellitus	32559	10631
E15-E16	Other disorders of glucose regulation and pancreatic internal secretion	3004	N/A
E20-E35	Disorders of other endocrine glands	5605	N/A
E50-E64	Other nutritional deficiencies	8163	N/A
E65-E68	Obesity and other hyperalimentation	26436	9278
E70-E90	Metabolic disorders	94662	6883
F00-F09	Organic, including symptomatic, mental disorders	10334	N/A
F10-F19	Mental and behavioural disorders due to psychoactive substance use	22731	9989
F30-F39	Mood [affective] disorders	22240	8967
F40-F48	Neurotic, stress-related and somatoform disorders	17716	7484
G20-G26	Extrapyramidal and movement disorders	6326	N/A
G30-G32	Other degenerative diseases of the nervous system	4574	N/A
G35-G37	Demyelinating diseases of the central nervous system	2338	N/A
G40-G47	Episodic and paroxysmal disorders	23315	7064
G50-G59	Nerve, nerve root and plexus disorders	19935	12054
G60-G64	Polyneuropathies and other disorders of the peripheral nervous system	4997	N/A
G80-G83	Cerebral palsy and other paralytic syndromes	5527	N/A
G90-G99	Other disorders of the nervous system	8344	N/A
H00-H06	Disorders of eyelid, lacrimal system and orbit	11979	6310
H15-H22	Disorders of sclera, cornea, iris and ciliary body	5325	N/A
H25-H28	Disorders of lens	38134	22042
H30-H36	Disorders of choroid and retina	14032	6025
H40-H42	Glaucoma	8993	4512
H43-H45	Disorders of vitreous body and globe	3131	N/A
H49-H52	Disorders of ocular muscles, binocular movement, accommodation and refraction	7655	4150
H53-H54	Visual disturbances and blindness	8224	N/A
H55-H59	Other disorders of eye and adnexa	2279	N/A
H65-H75	Diseases of middle ear and mastoid	2953	N/A
H80-H83	Diseases of inner ear	3343	N/A
H90-H95	Other disorders of ear	11651	3648
I05-I09	Chronic rheumatic heart diseases	7952	N/A
I10-I15	Hypertensive diseases	104878	46359
I20-I25	Ischaemic heart diseases	45574	11806
I26-I28	Pulmonary heart disease and diseases of pulmonary circulation	9255	N/A
I30-I52	Other forms of heart disease	53510	16853
I60-I69	Cerebrovascular diseases	18908	3043
I70-I79	Diseases of arteries, arterioles and capillaries	15707	2906
I80-I89	Diseases of veins, lymphatic vessels and lymph nodes, not elsewhere classified	35186	23394

Table S1. Disease Categories and Diagnosis Counts in RD and NRD Groups (Part 2)

ICD-10 Range	Description	Diagnoses in RD	Diagnoses in NRD
I95-I99	Other and unspecified disorders of the circulatory system	16730	N/A
J00-J06	Acute upper respiratory infections	3836	N/A
J09-J18	Influenza and pneumonia	24859	5840
J20-J22	Other acute lower respiratory infections	14387	3522
J30-J39	Other diseases of upper respiratory tract	16813	8882
J40-J47	Chronic lower respiratory diseases	43192	22044
J60-J70	Lung diseases due to external agents	3776	N/A
J80-J84	Other respiratory diseases principally affecting the interstitium	4849	N/A
J90-J94	Other diseases of pleura	16356	2928
J95-J99	Other diseases of the respiratory system	17218	2533
K00-K14	Diseases of oral cavity, salivary glands and jaws	17725	9903
K20-K31	Diseases of oesophagus, stomach and duodenum	80037	33693
K35-K38	Diseases of appendix	2930	2905
K40-K46	Hernia	55937	28998
K50-K52	Noninfective enteritis and colitis	20699	9878
K55-K64	Other diseases of intestines	86754	50222
K65-K67	Diseases of peritoneum	7572	2369
K70-K77	Diseases of liver	13307	N/A
K80-K87	Disorders of gallbladder, biliary tract and pancreas	24020	10720
K90-K93	Other diseases of the digestive system	21033	7390
L00-L08	Infections of the skin and subcutaneous tissue	14917	6768
L20-L30	Dermatitis and eczema	6638	2448
L40-L45	Papulosquamous disorders	5022	N/A
L55-L59	Radiation-related disorders of the skin and subcutaneous tissue	4500	N/A
L60-L75	Disorders of skin appendages	9021	7099
L80-L99	Other disorders of the skin and subcutaneous tissue	24540	9370
M05-M14	Inflammatory polyarthropathies	34385	13619
M15-M19	Arthrosis	58580	34461
M20-M25	Other joint disorders	35323	26258
M30-M36	Systemic connective tissue disorders	7382	N/A
M40-M43	Deforming dorsopathies	5812	N/A
M45-M49	Spondylopathies	21864	8024
M50-M54	Other dorsopathies	29000	13850
M60-M63	Disorders of muscles	2124	N/A
M65-M68	Disorders of synovium and tendon	8961	6887
M70-M79	Other soft tissue disorders	34444	18277
M80-M85	Disorders of bone density and structure	19136	6411
M86-M90	Other osteopathies	4770	N/A
N00-N08	Glomerular diseases	2987	N/A
N10-N16	Renal tubulo-interstitial diseases	7107	2406
N17-N19	Renal failure	30542	5742
N20-N23	Urolithiasis	7373	4100
N25-N29	Other disorders of kidney and ureter	6496	N/A
N30-N39	Other diseases of urinary system	41631	13176
N40-N51	Diseases of male genital organs	26971	11727
N60-N64	Disorders of breast	4851	3400
N70-N77	Inflammatory diseases of female pelvic organs	5186	3979
N80-N98	Noninflammatory disorders of female genital tract	33498	34767
O00-O08	Pregnancy with abortive outcome	N/A	3354
O20-O29	Other maternal disorders predominantly related to pregnancy	N/A	2626
O30-O48	Maternal care related to the fetus and amniotic cavity and possible delivery problems	2628	5852
O60-O75	Complications of labour and delivery	3343	8461
O80-O84	Delivery	N/A	2549
Q20-Q28	Congenital malformations of the circulatory system	2410	N/A
R00-R09	Symptoms and signs involving the circulatory and respiratory systems	65457	29136
R10-R19	Symptoms and signs involving the digestive system and abdomen	69886	38549
R20-R23	Symptoms and signs involving the skin and subcutaneous tissue	11101	3862
R25-R29	Symptoms and signs involving the nervous and musculoskeletal systems	21316	3510
R30-R39	Symptoms and signs involving the urinary system	38476	16035
R40-R46	Symptoms and signs involving cognition, perception, emotional state and behaviour	20890	5485
R47-R49	Symptoms and signs involving speech and voice	5245	N/A
R50-R69	General symptoms and signs	67545	27920
R70-R79	Abnormal findings on examination of blood, without diagnosis	17276	4726

Table S1. Disease Categories and Diagnosis Counts in RD and NRD Groups (Part 3)

ICD-10 Range	Description	Diagnoses in RD	Diagnoses in NRD
R90-R94	Abnormal findings on diagnostic imaging and in function studies, without diagnosis	20440	5247
S00-S09	Injuries to the head	13530	6427
S20-S29	Injuries to the thorax	4384	N/A
S30-S39	Injuries to the abdomen, lower back, lumbar spine and pelvis	4509	N/A
S40-S49	Injuries to the shoulder and upper arm	4832	3070
S50-S59	Injuries to the elbow and forearm	7211	6696
S60-S69	Injuries to the wrist and hand	6840	6474
S70-S79	Injuries to the hip and thigh	5693	2671
S80-S89	Injuries to the knee and lower leg	8469	6763
S90-S99	Injuries to the ankle and foot	2275	N/A
T36-T50	Poisoning by drugs, medicaments and biological substances	4111	N/A
T80-T88	Complications of surgical and medical care, not elsewhere classified	32337	11641
T90-T98	Sequelae of injuries, of poisoning and of other consequences of external causes	2801	N/A
U00-U49	Provisional assignment of new diseases of uncertain etiology or emergency use	3839	N/A
U82-U85	Resistance to antimicrobial and antineoplastic drugs	3163	N/A
W00-W19	Falls	27452	16007
W20-W49	Exposure to inanimate mechanical forces	5278	4691
X58-X59	Accidental exposure to other and unspecified factors	4382	2643
X60-X84	Intentional self-harm	2854	N/A
Y40-Y59	Drugs, medicaments and biological substances causing adverse effects in therapeutic use	15109	2466
Y83-Y84	Surgical and other medical procedures as the cause of abnormal reaction of the patient, or of later complication, without mention of misadventure at the time of the procedure	29729	9608
Y90-Y98	Supplementary factors related to causes of morbidity and mortality classified elsewhere	5776	N/A
Z00-Z13	Persons encountering health services for examination and investigation	66043	33973
Z20-Z29	Persons with potential health hazards related to communicable diseases	5403	N/A
Z30-Z39	Persons encountering health services in circumstances related to reproduction	7713	17863
Z40-Z54	Persons encountering health services for specific procedures and health care	84019	32054
Z55-Z65	Persons with potential health hazards related to socioeconomic and psychosocial circumstances	7632	N/A
Z70-Z76	Persons encountering health services in other circumstances	28113	10439

Table S2. Summary diagnostic information broken down by age, sex and ethnicity for RD and NRD groups.

**Group (RD)**	**No. of Records**	**Median No. of Diagnoses**	**Median Charlson Comorbidity Index**
Female	107,477	17	3
Male	104,389	18	4
Age 37-44	12,183	13	0
Age 45-52	32417	13	1
Age 53-60	59,557	15	3
Age 61-68	95,775	20	4
Age 69-73	11,934	23	5
White	199,627	17	3
Asian / Asian British	4,464	18	3
Black / Black British	3,190	18	3
Mixed	1,062	17	3
Other ethnicity	3,523	18	3
**Group (NRD)**			
Female	133,747	5	1
Male	94,361	5	1
Age 37-44	29,499	5	0
Age 45-52	59,582	4	0
Age 53-60	67,487	5	1
Age 61-68	65,805	6	2
Age 69-73	5,735	8	2
White	214,758	5	1
Asian / Asian British	4,087	5	1
Black / Black British	3,774	5	1
Mixed	1,473	5	1
Other ethnicity	4,016	5	1

Table S3. Top diagnoses for the RD group, broken down by age, sex and ethnicity.

Group (RD)	Top 5 Diagnoses (No. of Diagnoses)
**Female**	I10: Essential (Primary) hypertension (47,277)<br>Z86.4: Personal history of psychoactive substance abuse (21,796)<br>K44.9: Diaphragmatic hernia without obstruction or gangrene (20,959)<br>K57.3: Diverticular disease of large intestine without perforation or abscess (18,192)<br>J45.9: Asthma, unspecified (16,868)
**Male**	I10: Essential (Primary) hypertension (57,448)<br>Z86.4: Personal history of psychoactive substance abuse (32,712)<br>Z92.2: Personal history of long-term (current) use of other medicaments (21,463)<br>I25.1: Atherosclerotic heart disease (20,978)<br>N40: Hyperplasia of prostate (19,401)
**Age 37-44**	I10: Essential (Primary) hypertension (2,816)<br>Z37.0: Single live birth (2,449)<br>J45.9: Asthma, unspecified (1,849)<br>Z86.4: Personal history of psychoactive substance abuse (1,795)<br>F32.9: Depressive episode, unspecified (1,727)
**Age 45-52**	I10: Essential (Primary) hypertension (10,867)<br>Z86.4: Personal history of psychoactive substance abuse (5,832)<br>J45.9: Asthma, unspecified (4,812)<br>K44.9: Diaphragmatic hernia without obstruction or gangrene (4,683)<br>F32.9: Depressive episode, unspecified (4,265)
**Age 53-60**	I10: Essential (Primary) hypertension (27,415)<br>Z86.4: Personal history of psychoactive substance abuse (14,289)<br>K44.9: Diaphragmatic hernia without obstruction or gangrene (10,526)<br>K57.3: Diverticular disease of large intestine without perforation or abscess (9,303)<br>Z92.2: Personal history of long-term (current) use of other medicaments (8,849)
**Age 61-68**	I10: Essential (Primary) hypertension (55,775)<br>Z86.4: Personal history of psychoactive substance abuse (28,865)<br>Z92.2: Personal history of long-term (current) use of other medicaments (19,666)<br>K57.3: Diverticular disease of large intestine without perforation or abscess (19,466)<br>K44.9: Diaphragmatic hernia without obstruction or gangrene (18,815)
**Age 69-73**	I10: Essential (Primary) hypertension (7,852)<br>Z86.4: Personal history of psychoactive substance abuse (3,727)<br>Z92.2: Personal history of long-term (current) use of other medicaments (2,947)<br>Z86.7 Personal history of diseases of the circulatory system (2,729)<br>K57.3: Diverticular disease of large intestine without perforation or abscess (2,690)
**White**	I10: Essential (Primary) hypertension (97,503)<br>Z86.4: Personal history of psychoactive substance abuse (52,353)<br>K44.9: Diaphragmatic hernia without obstruction or gangrene (36,183)<br>K57.3: Diverticular disease of large intestine without perforation or abscess (34,461)<br>Z92.2: Personal history of long-term (current) use of other medicaments (33,367)
**Asian / Asian British**	I10: Essential (Primary) hypertension (2,799)<br>E11.9: Non-insulin-dependent diabetes mellitus without complications (1,787)<br>I25.1: Atherosclerotic heart disease (1,116)<br>R07.4: Chest pain, unspecified (1,055)<br>Z92.2: Personal history of long-term (current) use of other medicaments (1,006)
**Black / Black British**	I10: Essential (Primary) hypertension (2,017)<br>E11.9: Non-insulin-dependent diabetes mellitus without complications (992)<br>R07.4: Chest pain, unspecified (625)<br>E66.9: Obesity, unspecified (604)<br>Z86.7 Personal history of diseases of the circulatory system (518)
**Mixed**	I10: Essential (Primary) hypertension (500)<br>Z86.4: Personal history of psychoactive substance abuse (261)<br>J45.9: Asthma, unspecified (199)<br>E11.9: Non-insulin-dependent diabetes mellitus without complications (190)<br>K44.9: Diaphragmatic hernia without obstruction or gangrene (36,183)

Table S3. Top diagnoses for the RD group, broken down by age, sex and ethnicity (Continued).

**Other ethnicity**	I10: Essential (Primary) hypertension (1,906)<br>E11.9: Non-insulin-dependent diabetes mellitus without complications (875)<br>Z86.4: Personal history of psychoactive substance abuse (783)<br>R07.4: Chest pain, unspecified (598)<br>J45.9: Asthma, unspecified (574)

Table S4. Top diagnoses for the NRD group, broken down by age, sex and ethnicity.

Group (RD)	Top 5 Diagnoses (No. of Diagnoses)
**Female**	I10: Essential (Primary) hypertension (24,439)<br>K57.3: Diverticular disease of large intestine without perforation or abscess (11,867)<br>J45.9: Asthma, unspecified (11,067)<br>Z37.0: Single live birth (10,370)<br>K44.9: Diaphragmatic hernia without obstruction or gangrene (9,242)
**Male**	I10: Essential (Primary) hypertension (21,847)<br>K409: Unilateral or unspecified inguinal hernia, without obstruction or gangrene (9,464)<br>Z86.4: Personal history of psychoactive substance abuse (9,462)<br>K57.3: Diverticular disease of large intestine without perforation or abscess (9,028)<br>N40: Hyperplasia of prostate (6,623)
**Age 37-44**	Z37.0: Single live birth (6,466)<br>J45.9: Asthma, unspecified (2,361)<br>I10: Essential (Primary) hypertension (2,193)<br>O70.1: Second degree perineal laceration during delivery (2,089)<br>Z30.2: Contraceptive management: Sterilisation (2,074)
**Age 45-52**	I10: Essential (Primary) hypertension (7,343)<br>Z12.1: Special screening examination for neoplasm of intestinal tract (5,269)<br>J45.9: Asthma, unspecified (4,623)<br>K57.3: Diverticular disease of large intestine without perforation or abscess (4,323)<br>Z37.0: Single live birth (3,751)
**Age 53-60**	I10: Essential (Primary) hypertension (13,936)<br>K57.3: Diverticular disease of large intestine without perforation or abscess (6,409)<br>Z86.4: Personal history of psychoactive substance abuse (5,608)<br>J45.9: Asthma, unspecified (5,052)<br>K44.9: Diaphragmatic hernia without obstruction or gangrene (4,559)
**Age 61-68**	I10: Essential (Primary) hypertension (20,643)<br>K57.3: Diverticular disease of large intestine without perforation or abscess (8,271)<br>H26.9: Cataract, unspecified (7,277)<br>Z86.4: Personal history of psychoactive substance abuse (7,192)<br>K44.9: Diaphragmatic hernia without obstruction or gangrene (5,716)
**Age 69-73**	I10: Essential (Primary) hypertension (2,171)<br>H26.9: Cataract, unspecified (1,058)<br>K57.3: Diverticular disease of large intestine without perforation or abscess (824)<br>Z86.4: Personal history of psychoactive substance abuse (673)<br>K44.9: Diaphragmatic hernia without obstruction or gangrene (587)
**White**	I10: Essential (Primary) hypertension (43,186)<br>K57.3: Diverticular disease of large intestine without perforation or abscess (20,526)<br>Z86.4: Personal history of psychoactive substance abuse (17,870)<br>J45.9: Asthma, unspecified (16,390)<br>K44.9: Diaphragmatic hernia without obstruction or gangrene (14,082)
**Asian / Asian British**	I10: Essential (Primary) hypertension (980)<br>E11.9: Non-insulin-dependent diabetes mellitus without complications (558)<br>H26.9: Cataract, unspecified (393)<br>J45.9: Asthma, unspecified (365)<br>R07.4: Chest pain, unspecified (331)
**Black / Black British**	I10: Essential (Primary) hypertension (1,062)<br>D25.9: Leiomyoma of uterus, unspecified (456)<br>E11.9: Non-insulin-dependent diabetes mellitus without complications (361)<br>J45.9: Asthma, unspecified (350)<br>Z37.0: Single live birth (341)
**Mixed**	I10: Essential (Primary) hypertension (237)<br>J45.9: Asthma, unspecified (147)<br>Z37.0: Single live birth (146)<br>Z86.4: Personal history of psychoactive substance abuse (116)<br>F17.1: Mental and behavioural disorders due to use of tobacco: harmful use (111)

Table S4. Top diagnoses for the NRD group, broken down by age, sex and ethnicity (continued).

**Other ethnicity**	I10: Essential (Primary) hypertension (821)<br>J45.9: Asthma, unspecified (302)<br>E11.9: Non-insulin-dependent diabetes mellitus without complications (289)<br>Z37.0: Single live birth (289)<br>H26.9: Cataract, unspecified (272)

Table S5. RD Cluster 0 Relative Risk and Phi Coefficient Results

**Field 1**	**Field 2**	**Relative Risk Field1 ->Field2 (CI)**	**Relative Risk Field2 ->Field1 (CI)**	**Phi Coefficient**
Melanoma and other malignant neoplasms of skin	Disorders of lens	1.066; (1.036 - 1.097)	1.073; (1.039 - 1.107)	0.009
Melanoma and other malignant neoplasms of skin	Disorders of sclera, cornea, iris and ciliary body	0.785; (0.713 - 0.865)	0.799; (0.730 - 0.874)	-0.011
Melanoma and other malignant neoplasms of skin	Disorders of choroid and retina	0.966; (0.916 - 1.019)	0.967; (0.919 - 1.018)	-0.003
Melanoma and other malignant neoplasms of skin	Disorders of ocular muscles, binocular movement, accommodation and refraction	0.983; (0.914 - 1.058)	0.985; (0.920 - 1.054)	-0.001
Disorders of lens	Disorders of sclera, cornea, iris and ciliary body	21.252; (19.824 - 22.784)	5.040; (4.961 - 5.120)	0.269
Disorders of lens	Disorders of choroid and retina	9.638; (9.319 - 9.968)	4.696; (4.623 - 4.770)	0.346
Disorders of lens	Disorders of ocular muscles, binocular movement, accommodation and refraction	22.168; (20.908 - 23.504)	5.330; (5.254 - 5.407)	0.327
Disorders of sclera, cornea, iris and ciliary body	Disorders of choroid and retina	4.771; (4.559 - 4.993)	5.721; (5.405 - 6.055)	0.144
Disorders of sclera, cornea, iris and ciliary body	Disorders of ocular muscles, binocular movement, accommodation and refraction	14.328; (13.732 - 14.951)	16.898; (16.071 - 17.768)	0.303
Disorders of choroid and retina	Disorders of ocular muscles, binocular movement, accommodation and refraction	5.365; (5.118 - 5.624)	4.721; (4.535 - 4.915)	0.163

Table S6. RD Cluster 1 Relative Risk and Phi Coefficient Results

**Field 1**	**Field 2**	**Relative Risk Field1 ->Field2 (CI)**	**Relative Risk Field2 ->Field1 (CI)**	**Phi Coefficient**
Metabolic disorders	Hypertensive diseases	2.093; (2.074 - 2.112)	2.336; 2.311 - 2.362)	0.361

Table S7. RD Cluster 2 Relative Risk and Phi Coefficient Results

Field 1	Field 2	Relative Risk Field1 ->Field2 (CI)	Relative Risk Field2 ->Field1 (CI)	Phi Coefficient
Hernia	Diseases of oesophagus, stomach and duodenum	2.546; (2.521 - 2.571)	3.549; (3.494 - 3.604)	0.377
Hernia	Symptoms and signs involving the digestive system and abdomen	1.535; (1.516 - 1.553)	1.620; (1.597 - 1.643)	0.145
Hernia	Other diseases of intestines	1.481 (1.466 - 1.496)	1.679; (1.656 - 1.703)	0.157
Hernia	Arthrosis	1.379; (1.360 - 1.399)	1.388; (1.368 - 1.409)	0.094
Hernia	Other joint disorders	1.306; (1.280 - 1.332)	1.261; (1.239 - 1.283)	0.056
Diseases of oesophagus, stomach and duodenum	Symptoms and signs involving the digestive system and abdomen	1.995; (1.971 - 2.019)	1.863; (1.843 - 1.882)	0.246
Diseases of oesophagus, stomach and duodenum	Other diseases of intestines	1.581; (1.565 - 1.597)	1.632; (1.614 - 1.650)	0.192
Diseases of oesophagus, stomach and duodenum	Arthrosis	1.470; (1.450 - 1.490)	1.379; (1.364 - 1.394)	0.12
Diseases of oesophagus, stomach and duodenum	Other joint disorders	1.427; (1.400 - 1.455)	1.288; (1.271 - 1.304)	0.08
Symptoms and signs involving the digestive system and abdomen	Other diseases of intestines	1.784; (1.766 - 1.801)	1.994; (1.970 - 2.019)	0.244
Symptoms and signs involving the digestive system and abdomen	Arthrosis	1.360; (1.341 - 1.379)	1.325; (1.308 - 1.341)	0.093
Symptoms and signs involving the digestive system and abdomen	Other joint disorders	1.432; (1.405 - 1.460)	1.320; (1.302 - 1.339)	0.08
Other diseases of intestines	Arthrosis	1.433; (1.413 - 1.453)	1.327; (1.314 - 1.341)	0.112
Other diseases of intestines	Other joint disorders	1.357; (1.332 - 1.383)	1.229; (1.214 - 1.244)	0.069
Arthrosis	Other joint disorders	4.368; (4.284 - 4.453)	3.025; (2.989 - 3.063)	0.349

Table S8. RD Cluster 3 Relative Risk and Phi Coefficient Results

Field 1	Field 2	Relative Risk Field1 ->Field2 (CI)	Relative Risk Field2 ->Field1 (CI)	Phi Coefficient
Ischaemic heart diseases	Metabolic disorders	2.101; (2.084 - 2.118)	3.899; (3.823 - 3.977)	0.329
Ischaemic heart diseases	Hypertensive diseases	1.905; (1.891 - 1.919)	3.820; (3.741 - 3.901)	0.308
Ischaemic heart diseases	Other forms of heart disease	3.296; (3.252 - 3.341)	3.725; (3.667 - 3.784)	0.367
Ischaemic heart diseases	Symptoms and signs involving the circulatory and respiratory systems	2.298; (2.271 - 2.326)	2.790; (2.746 - 2.835)	0.279
Ischaemic heart diseases	Renal failure	2.302; (2.255 - 2.350)	2.078; (2.042 - 2.113)	0.172
Ischaemic heart diseases	Diabetes mellitus	2.321; (2.276 - 2.367)	2.118; (2.083 - 2.154)	0.18
Metabolic disorders	Hypertensive diseases	2.093; (2.074 - 2.112)	2.336; (2.311 - 2.362)	0.361
Metabolic disorders	Other forms of heart disease	2.355; (2.318 - 2.392)	1.742; (1.726 - 1.757)	0.244
Metabolic disorders	Symptoms and signs involving the circulatory and respiratory systems	1.979; (1.953 - 2.006)	1.656; (1.641 - 1.671)	0.226
Metabolic disorders	Renal failure	3.285; (3.208 - 3.363)	1.817; (1.801 - 1.833)	0.231
Metabolic disorders	Diabetes mellitus	2.856; (2.793 - 2.919)	1.738; (1.723 - 1.754)	0.215
Hypertensive diseases	Other forms of heart disease	2.630; (2.586 - 2.675)	1.720; (1.707 - 1.734)	0.262
Hypertensive diseases	Symptoms and signs involving the circulatory and respiratory systems	1.834; (1.809 - 1.859)	1.497; (1.485 - 1.510)	0.197
Hypertensive diseases	Renal failure	3.345; (3.262 - 3.430)	1.706; (1.692 - 1.719)	0.223
Hypertensive diseases	Diabetes mellitus	4.144; (4.038 - 4.253)	1.827; (1.813 - 1.841)	0.262
Other forms of heart disease	Symptoms and signs involving the circulatory and respiratory systems	2.145; (2.120 - 2.171)	2.366; (2.333 - 2.400)	0.258
Other forms of heart disease	Renal failure	3.241; (3.176 - 3.307)	2.524; (2.489 - 2.560)	0.255
Other forms of heart disease	Diabetes mellitus	1.884; (1.847 - 1.923)	1.708; (1.681 - 1.736)	0.134
Symptoms and signs involving the circulatory and respiratory systems	Renal failure	2.086; (2.044 - 2.129)	1.725; (1.702 - 1.749)	0.154
Symptoms and signs involving the circulatory and respiratory systems	Diabetes mellitus	1.723; (1.689 - 1.758)	1.521; (1.500 - 1.543)	0.116
Renal failure	Diabetes mellitus	2.518; (2.467 - 2.570)	2.562; (2.509 - 2.616)	0.186

Table S9. RD Cluster 4 Relative Risk and Phi Coefficient Results

**Field 1**	**Field 2**	**Relative Risk Field1 ->Field2 (CI)**	**Relative Risk Field2 ->Field1 (CI)**	**Phi Coefficient**
Diseases of oesophagus, stomach and duodenum	Symptoms and signs involving the digestive system and abdomen	1.995; (1.971 - 2.019)	1.863; (1.843 - 1.882)	0.246
Diseases of oesophagus, stomach and duodenum	Other diseases of intestines	1.581; (1.565 - 1.597)	1.632; (1.614 - 1.650)	0.192
Diseases of oesophagus, stomach and duodenum	Noninflammatory disorders of female genital tract	1.227; (1.203 - 1.252)	1.158; (1.143 - 1.175)	0.044
Symptoms and signs involving the digestive system and abdomen	Other diseases of intestines	1.784; (1.766 - 1.801)	1.994; (1.970 - 2.019)	0.244
Symptoms and signs involving the digestive system and abdomen	Noninflammatory disorders of female genital tract	1.585; (1.554 - 1.616)	1.416; (1.396 - 1.436)	0.1
Other diseases of intestines	Noninflammatory disorders of female genital tract	1.288; (1.263 - 1.313)	1.186; (1.171 - 1.201)	0.055

Table S10. RD Cluster 6 Relative Risk and Phi Coefficient Results

**Field 1**	**Field 2**	**Relative Risk Field1 ->Field2 (CI)**	**Relative Risk Field2 ->Field1 (CI)**	**Phi Coefficient**
Other diseases of intestines	Benign neoplasms	1.848; (1.820 - 1.876)	1.558; (1.542 - 1.573)	0.175
Other diseases of intestines	Arthrosis	1.433; (1.413 - 1.453)	1.327; (1.314 - 1.341)	0.112
Other diseases of intestines	Other joint disorders	1.357; (1.332 - 1.383)	1.229; (1.214 - 1.244)	0.069
Benign neoplasms	Arthrosis	1.096; (1.079 - 1.113)	1.101; (1.083 - 1.119)	0.025
Benign neoplasms	Other joint disorders	1.088; (1.065 - 1.112)	1.079 (1.058 - 1.100)	0.017
Arthrosis	Other joint disorders	4.368; (4.284 - 4.453)	3.025; (2.989 - 3.063)	0.349

Table S11. RD Cluster 7 Relative Risk and Phi Coefficient Results

Field 1	Field 2	Relative Risk Field1 ->Field2 (CI)	Relative Risk Field2 ->Field1 (CI)	Phi Coefficient
Persons encountering health services in circumstances related to reproduction	Complications of labour and delivery	1675.163; (1274.958 - 2200.990)	46.422; (45.074 - 47.811)	0.641
Persons encountering health services in circumstances related to reproduction	Maternal care related to the fetus and amniotic cavity and possible delivery problems	1152.510; (891.243 - 1490.367)	39.763; (38.680 - 40.876)	0.563
Persons encountering health services in circumstances related to reproduction	Noninflammatory disorders of female genital tract	2.524; (2.448 - 2.602)	3.237; (3.096 - 3.384)	0.117
Persons encountering health services in circumstances related to reproduction	Benign neoplasms	1.172; (1.130 - 1.215)	1.230; (1.171 - 1.291)	0.018
Persons encountering health services in circumstances related to reproduction	Disorders of breast	2.122; (1.910 - 2.356)	2.089; (1.887 - 2.313)	0.031
Complications of labour and delivery	Maternal care related to the fetus and amniotic cavity and possible delivery problems	181.196; (167.155 - 196.417)	112.143; (105.926 - 118.725)	0.655
Complications of labour and delivery	Noninflammatory disorders of female genital tract	2.400; (2.294 - 2.511)	3.144; (2.934 - 3.369)	0.074
Complications of labour and delivery	Benign neoplasms	1.159; (1.098 - 1.225)	1.218; (1.130 - 1.312)	0.011
Complications of labour and delivery	Disorders of breast	2.404; (2.079 - 2.779)	2.429; (2.096 - 2.814)	0.026
Maternal care related to the fetus and amniotic cavity and possible delivery problems	Noninflammatory disorders of female genital tract	2.523; (2.403 - 2.649)	3.427; (3.171 - 3.703)	0.072
Maternal care related to the fetus and amniotic cavity and possible delivery problems	Benign neoplasms	1.154; (1.085 - 1.228)	1.211; (1.113 - 1.318)	0.01
Maternal care related to the fetus and amniotic cavity and possible delivery problems	Disorders of breast	2.279; (1.929 - 2.693)	2.311; (1.948 - 2.741)	0.021
Noninflammatory disorders of female genital tract	Benign neoplasms	1.744; (1.716 - 1.773)	1.903; (1.866 - 1.941)	0.138
Noninflammatory disorders of female genital tract	Disorders of breast	2.829; (2.669 - 2.998)	2.257; (2.169 - 2.349)	0.079
Benign neoplasms	Disorders of breast	1.707; (1.611 - 1.809)	1.472; (1.416 - 1.530)	0.04

Table S12. RD Cluster 8 Relative Risk and Phi Coefficient Results

**Field 1**	**Field 2**	**Relative Risk Field1 ->Field2 (CI)**	**Relative Risk Field2 ->Field1 (CI)**	**Phi Coefficient**
Malignant neoplasms of male genital organs	Other diseases of urinary system	1.387; (1.348 - 1.427)	1.479; (1.427 - 1.533)	0.047
Malignant neoplasms of male genital organs	Symptoms and signs involving the urinary system	1.873; (1.825 - 1.922)	2.133; (2.063 - 2.206)	0.097
Malignant neoplasms of male genital organs	Diseases of male genital organs	2.747; (2.673 - 2.823)	3.123; (3.022 - 3.228)	0.149
Malignant neoplasms of male genital organs	Persons encountering health services for specific procedures and health care	1.410; (1.388 - 1.433)	1.817; (1.760 - 1.875)	0.081
Malignant neoplasms of male genital organs	Abnormal findings on examination of blood, without diagnosis	2.640; (2.544 - 2.740)	2.710; (2.607 - 2.816)	0.11
Malignant neoplasms of male genital organs	Malignant neoplasms of urinary tract	2.107; (1.954 - 2.271)	2.014; (1.880 - 2.158)	0.043
Other diseases of urinary system	Symptoms and signs involving the urinary system	3.990; (3.923 - 4.058)	3.784; (3.725 - 3.844)	0.353
Other diseases of urinary system	Diseases of male genital organs	3.054; (2.989 - 3.121)	2.627; (2.582 - 2.672)	0.222
Other diseases of urinary system	Persons encountering health services for specific procedures and health care	1.578; (1.562 - 1.595)	1.953; (1.919 - 1.987)	0.167
Other diseases of urinary system	Abnormal findings on examination of blood, without diagnosis	1.838; (1.783 - 1.895)	1.663; (1.624 - 1.704)	0.085
Other diseases of urinary system	Malignant neoplasms of urinary tract	4.769; (4.531 - 5.019)	2.877; (2.804 - 2.952)	0.143
Symptoms and signs involving the urinary system	Diseases of male genital organs	3.814; (3.734 - 3.896)	3.246; (3.190 - 3.302)	0.274
Symptoms and signs involving the urinary system	Persons encountering health services for specific procedures and health care	1.599; (1.582 - 1.616)	2.032; (1.996 - 2.070)	0.169
Symptoms and signs involving the urinary system	Abnormal findings on examination of blood, without diagnosis	2.188; (2.123 - 2.254)	1.937; (1.892 - 1.983)	0.112
Symptoms and signs involving the urinary system	Malignant neoplasms of urinary tract	3.598; (3.418 - 3.788)	2.546; (2.469 - 2.625)	0.113
Diseases of male genital organs	Persons encountering health services for specific procedures and health care	1.353; (1.335 - 1.371)	1.606; (1.570 - 1.642)	0.091
Diseases of male genital organs	Abnormal findings on examination of blood, without diagnosis	2.634; (2.555 - 2.717)	2.436; (2.371 - 2.503)	0.134
Diseases of male genital organs	Malignant neoplasms of urinary tract	3.541; (3.356 - 3.735)	2.804; (2.700 - 2.913)	0.106
Persons encountering health services for specific procedures and health care	Abnormal findings on examination of blood, without diagnosis	2.142; (2.081 - 2.205)	1.539; (1.518 - 1.561)	0.115
Persons encountering health services for specific procedures and health care	Malignant neoplasms of urinary tract	2.538; (2.406 - 2.676)	1.602; (1.569 - 1.635)	0.077
Abnormal findings on examination of blood, without diagnosis	Malignant neoplasms of urinary tract	1.590; (1.472 - 1.719)	1.539; (1.434 - 1.652)	0.026

Table S13. RD Cluster 9 Relative Risk and Phi Coefficient Results

Field 1	Field 2	Relative Risk Field1 ->Field2 (CI)	Relative Risk Field2 ->Field1 (CI)	Phi Coefficient
Benign neoplasms	Noninflammatory disorders of female genital tract	1.903; (1.866 - 1.941)	1.744; (1.716 - 1.773)	0.138
Benign neoplasms	Melanoma and other malignant neoplasms of skin	1.175; (1.142 - 1.208)	1.144; (1.118 - 1.170)	0.024
Benign neoplasms	Inflammatory diseases of female pelvic organs	2.646; (2.508 - 2.792)	1.933; (1.875 - 1.993)	0.08
Benign neoplasms	Malignant neoplasms of female genital organs	1.545; (1.455 - 1.640)	1.376; (1.320 - 1.434)	0.031
Noninflammatory disorders of female genital tract	Melanoma and other malignant neoplasms of skin	0.733; (0.705 - 0.762)	0.746; (0.718 - 0.774)	-0.034
Noninflammatory disorders of female genital tract	Inflammatory diseases of female pelvic organs	12.491; (11.783 - 13.241)	4.853; (4.753 - 4.954)	0.236
Noninflammatory disorders of female genital tract	Malignant neoplasms of female genital organs	5.789; (5.474 - 6.123)	3.475; (3.375 - 3.578)	0.15
Melanoma and other malignant neoplasms of skin	Inflammatory diseases of female pelvic organs	0.625; (0.561 - 0.696)	0.645; (0.582 - 0.714)	-0.019
Melanoma and other malignant neoplasms of skin	Malignant neoplasms of female genital organs	0.579; (0.515 - 0.651)	0.600; (0.536 - 0.671)	-0.02
Inflammatory diseases of female pelvic organs	Malignant neoplasms of female genital organs	7.054; (6.543 - 7.605)	6.953; (6.457 - 7.487)	0.123

Table S14. RD Cluster 10 Relative Risk and Phi Coefficient Results

Field 1	Field 2	Relative Risk Field1 ->Field2 (CI)	Relative Risk Field2 ->Field1 (CI)	Phi Coefficien
Malignant neoplasms of ill-defined, secondary and unspecified sites	Persons encountering health services for specific procedures and health care	2.222; (2.202 - 2.243)	5.665; (5.484 - 5.852)	0.262
Malignant neoplasms of ill-defined, secondary and unspecified sites	Malignant neoplasms of digestive organs	9.628; (9.343 - 9.923)	7.458; (7.287 - 7.633)	0.366
Malignant neoplasms of ill-defined, secondary and unspecified sites	Other diseases of blood and blood-forming organs	4.795; (4.615 - 4.981)	4.025; (3.904 - 4.150)	0.185
Malignant neoplasms of ill-defined, secondary and unspecified sites	Other bacterial diseases	3.393; (3.285 - 3.505)	3.201; (3.107 - 3.299)	0.161
Malignant neoplasms of ill-defined, secondary and unspecified sites	Drugs, medicaments and biological substances causing adverse effects in therapeutic use	3.804; (3.684 - 3.928)	3.535; (3.434 - 3.639)	0.181
Malignant neoplasms of ill-defined, secondary and unspecified sites	General symptoms and signs	1.660; (1.635 - 1.686)	2.116; (2.062 - 2.171)	0.125
Malignant neoplasms of ill-defined, secondary and unspecified sites	Malignant neoplasms of respiratory and intrathoracic organs	15.357; (14.611 - 16.141)	7.669; (7.484 - 7.859)	0.305
Persons encountering health services for specific procedures and health care	Malignant neoplasms of digestive organs	3.488; (3.367 - 3.613)	1.853; (1.830 - 1.876)	0.161
Persons encountering health services for specific procedures and health care	Other diseases of blood and blood-forming organs	4.123; (3.951 - 4.303)	1.925; (1.900 - 1.950)	0.154
Persons encountering health services for specific procedures and health care	Other bacterial diseases	4.204; (4.061 - 4.352)	1.985; (1.963 - 2.007)	0.194
Persons encountering health services for specific procedures and health care	Drugs, medicaments and biological substances causing adverse effects in therapeutic use	3.409; (3.297 - 3.525)	1.849; (1.827 - 1.871)	0.167
Persons encountering health services for specific procedures and health care	General symptoms and signs	1.857; (1.834 - 1.880)	1.691; (1.674 - 1.708)	0.214
Persons encountering health services for specific procedures and health care	Malignant neoplasms of respiratory and intrathoracic organs	4.205; (3.975 - 4.448)	1.899; (1.869 - 1.930)	0.119
Malignant neoplasms of digestive organs	Other diseases of blood and blood-forming organs	2.278; (2.159 - 2.403)	2.229; (2.117 - 2.346)	0.066
Malignant neoplasms of digestive organs	Other bacterial diseases	2.433; (2.335 - 2.535)	2.465; (2.363 - 2.571)	0.091
Malignant neoplasms of digestive organs	Drugs, medicaments and biological substances causing adverse effects in therapeutic use	2.286; (2.190 - 2.387)	2.306; (2.208 - 2.409)	0.081
Malignant neoplasms of digestive organs	General symptoms and signs	1.379; (1.351 - 1.407)	1.605; (1.554 - 1.658)	0.062
Malignant neoplasms of digestive organs	Malignant neoplasms of respiratory and intrathoracic organs	1.068; (0.969 - 1.178)	1.066; (0.970 - 1.170)	0.003
Other diseases of blood and blood-forming organs	Other bacterial diseases	8.389; (8.157 - 8.628)	10.447; (10.086 - 10.822)	0.329
Other diseases of blood and blood-forming organs	Drugs, medicaments and biological substances causing adverse effects in therapeutic use	6.294; (6.099 - 6.495)	7.232; (6.971 - 7.503)	0.251
Other diseases of blood and blood-forming organs	General symptoms and signs	1.898; (1.864 - 1.931)	2.947; (2.837 - 3.061)	0.126
Other diseases of blood and blood-forming organs	Malignant neoplasms of respiratory and intrathoracic organs	2.476; (2.292 - 2.674)	2.403; (2.233 - 2.586)	0.051
Other bacterial diseases	Drugs, medicaments and biological substances causing adverse effects in therapeutic use	4.642; (4.496 - 4.794)	4.607; (4.463 - 4.756)	0.208
Other bacterial diseases	General symptoms and signs	2.183; (2.153 - 2.212)	3.804; (3.686 - 3.925)	0.194
Other bacterial diseases	Malignant neoplasms of respiratory and intrathoracic organs	2.244; (2.096 - 2.403)	2.123; (1.996 - 2.258)	0.051
Drugs, medicaments and biological substances causing adverse effects in therapeutic use	General symptoms and signs	2.067; (2.038 - 2.097)	3.376; (3.272 - 3.484)	0.175
Drugs, medicaments and biological substances causing adverse effects in therapeutic use	Malignant neoplasms of respiratory and intrathoracic organs	2.601; (2.437 - 2.777)	2.431; (2.293 - 2.577)	0.064
General symptoms and signs	Malignant neoplasms of respiratory and intrathoracic organs	2.221; (2.114 - 2.334)	1.628; (1.587 - 1.670)	0.07

Table S15. RD Cluster 11 Relative Risk and Phi Coefficient Results

Field 1	Field 2	Relative Risk Field1 ->Field2; (CI)	Relative Risk Field2 ->Field1; (CI)	Phi Coefficient
Intentional self-harm	Poisoning by drugs, medicaments and biological substances	133.685; (126.889 - 140.845)	677.902; (588.720 - 780.593)	0.772
Intentional self-harm	Mood [affective] disorders	7.464; (7.270 - 7.663)	22.006; (20.302 - 23.853)	0.235
Intentional self-harm	Mental and behavioural disorders due to psychoactive substance use	4.374; (4.191 - 4.564)	6.776; (6.302 - 7.285)	0.129
Intentional self-harm	Neurotic, stress-related and somatoform disorders	4.956; (4.725 - 5.199)	7.110; (6.606 - 7.652)	0.131
Intentional self-harm	Persons with potential health hazards related to socioeconomic and psychosocial circumstances	6.635; (6.171 - 7.133)	7.642; (7.018 - 8.322)	0.117
Intentional self-harm	Symptoms and signs involving cognition, perception, emotional state and behaviour	3.061; (2.887 - 3.245)	3.800; (3.510 - 4.114)	0.076
Poisoning by drugs, medicaments and biological substances	Mood [affective] disorders	6.028; (5.853 - 6.207)	11.607; (10.928 - 12.328)	0.216
Poisoning by drugs, medicaments and biological substances	Mental and behavioural disorders due to psychoactive substance use	3.814; (3.662 - 3.971)	5.275; (4.962 - 5.608)	0.128
Poisoning by drugs, medicaments and biological substances	Neurotic, stress-related and somatoform disorders	4.152; (3.964 - 4.348)	5.329; (5.002 - 5.677)	0.124
Poisoning by drugs, medicaments and biological substances	Persons with potential health hazards related to socioeconomic and psychosocial circumstances	5.614; (5.244 - 6.009)	6.099; (5.657 - 6.575)	0.113
Poisoning by drugs, medicaments and biological substances	Symptoms and signs involving cognition, perception, emotional state and behaviour	3.066; (2.918 - 3.222)	3.746; (3.508 - 4.001)	0.091
Mood [affective] disorders	Mental and behavioural disorders due to psychoactive substance use	2.653; (2.582 - 2.727)	2.665; (2.593 - 2.739)	0.15
Mood [affective] disorders	Neurotic, stress-related and somatoform disorders	7.304; (7.116 - 7.497)	6.368; (6.227 - 6.513)	0.351
Mood [affective] disorders	Persons with potential health hazards related to socioeconomic and psychosocial circumstances	3.486; (3.325 - 3.656)	2.960; (2.851 - 3.073)	0.117
Mood [affective] disorders	Symptoms and signs involving cognition, perception, emotional state and behaviour	2.720; (2.644 - 2.799)	2.688; (2.614 - 2.764)	0.148
Mental and behavioural disorders due to psychoactive substance use	Neurotic, stress-related and somatoform disorders	2.126; (2.056 - 2.200)	2.066; (2.001 - 2.134)	0.094
Mental and behavioural disorders due to psychoactive substance use	Persons with potential health hazards related to socioeconomic and psychosocial circumstances	3.080; (2.934 - 3.234)	2.670; (2.567 - 2.776)	0.102
Mental and behavioural disorders due to psychoactive substance use	Symptoms and signs involving cognition, perception, emotional state and behaviour	2.010; (1.948 - 2.074)	1.991; (1.931 - 2.053)	0.093
Neurotic, stress-related and somatoform disorders	Persons with potential health hazards related to socioeconomic and psychosocial circumstances	2.930; (2.779 - 3.089)	2.675; (2.555 - 2.801)	0.089
Neurotic, stress-related and somatoform disorders	Symptoms and signs involving cognition, perception, emotional state and behaviour	2.489; (2.412 - 2.568)	2.552; (2.470 - 2.637)	0.121
Persons with potential health hazards related to socioeconomic and psychosocial circumstances	Symptoms and signs involving cognition, perception, emotional state and behaviour	2.950; (2.836 - 3.067)	3.411; (3.250 - 3.580)	0.112
